# Structure‐guided optimization of SLY1 expression and purification in *Escherichia coli*

**DOI:** 10.1002/pro.70592

**Published:** 2026-04-20

**Authors:** Souleïmen Jmii, William Bouard, Mathilde Rochas, François Dragon, Laurent Cappadocia

**Affiliations:** ^1^ Département de chimie Université du Québec à Montréal Montréal Quebec Canada; ^2^ Centre Sève, Department of Biology Université de Sherbrooke Sherbrooke Quebec Canada; ^3^ PROTEO, Quebec Network for Research on Protein, Function, Engineering and Applications Montréal Quebec Canada; ^4^ Département des sciences biologiques Université du Québec à Montréal Montréal Quebec Canada; ^5^ Université Paris‐Saclay, Centre national de la recherche scientifique (CNRS) Institut national de recherche pour l'agriculture, l'alimentation et l'environnement (INRAE), Université Evry, Institute of Plant Sciences Paris‐Saclay (IPS2) Gif sur Yvette France; ^6^ Centre d'excellence en recherche sur les maladies orphelines – Fondation Courtois (CERMO‐FC) Montréal Quebec Canada

**Keywords:** ASK1, gibberellin signaling, plant E3 ligases, plant proteins, protein expression and purification, protein–protein interactions, SLY1, structure prediction

## Abstract

The SCF^SLY1^ ubiquitin ligase complex is a key regulator of gibberellin signaling, mediating the degradation of DELLA proteins through targeted ubiquitination. To enable detailed biochemical and structural studies of this complex, efficient recombinant production of SLY1 and its adaptor ASK1 is essential. In this study, we optimized the co‐expression and purification of the ASK1‐SLY1 complex in *Escherichia coli*, focusing on molecular determinants that influence solubility, stability, and protein interaction. Hydrophobicity analyses and AlphaFold structural modeling of ASK1 and SLY1 identified prominent hydrophobic surfaces, particularly around helix α7 of SLY1, which are involved in DELLA binding and potentially driving aggregation during purification. Based on these insights, we adopted a co‐expression strategy with an MBP tag fused at the N‐terminal end of one partner, which significantly enhanced solubility and enabled successful isolation of the intact ASK1‐SLY1 complex. Overall, this work presents an optimized protocol for recombinant production of the ASK1‐SLY1 complex and provides structural rationale for overcoming key challenges in its expression and purification.

## 1 INTRODUCTION

DELLA proteins are central repressors of plant growth, coordinating biological processes such as seed germination, stem and root elongation, flowering, and fruit development. DELLA degradation is mediated by ubiquitination, a post‐translational modification in which ubiquitin proteins are attached covalently to DELLA proteins. This triggers their degradation by the 26S proteasome and forces the release of growth modulating factors (Fukazawa et al., 2021; Qin et al., 2014; Tyler et al., 2004; Ubeda‐Tomás et al., 2008). The ubiquitination process relies on an ubiquitin E3 ligase, which selectively recognizes and recruits substrates for degradation (Hua & Vierstra, 2011). In plants, the SCF^SKP1‐CUL1‐F‐box^ complex represents a major class of ubiquitin E3 ligases, composed of four core components: the scaffold protein CULLIN1, the RING‐finger protein RBX that recruits an E2 ubiquitin‐conjugating enzyme, the adaptor protein Arabidopsis SKP1‐like (SKP1/ASK1), and an F‐box domain protein that confers substrate specificity (Zheng et al., 2002). In *Arabidopsis thaliana*, nearly 700 F‐box proteins have been identified, involved in a wide range of biological processes, particularly hormonal signaling (Gagne et al., 2002). SLEEPY1 (SLY1) and its paralog SNEEZY 1 (SNE1) form the SLY1 subfamily of F‐box proteins which target DELLA for degradation (Ariizumi et al., 2011). Knockout of *AtSLY1* leads to dwarfism through DELLA proteins accumulation, thereby sequestering growth factors and preventing growth stimulation (Ariizumi et al., 2011; Nelson & Steber, 2017). The cryogenic electron microscopy (cryo‐EM) structure of the GID1/RGA/SLY1/ASK1 complex indicates that hydrophobic interactions mediated by both the N‐terminal F‐box domain (aa 29–75) and the C‐terminal α‐helix (aa 123–142) of SLY1 are involved in its interaction with ASK1 and DELLA, respectively (Dahal et al., 2025; Hirano et al., 2010; Islam et al., 2025). Reconstruction of the ancestral form of SLY1 from *Marchantia polymorpha* (Ji et al., 2023) further suggested an increase in both the rigidity and hydrophobicity of the last α‐helix (α‐helix 7) of SLY1 during evolution. This helix contains the GGF an LSL motifs essential for DELLA recognition (Gomi et al., 2004) and the shift refined the ancestral broad promiscuity of SLY1 into highly specific recognition of DELLA members, favoring tighter regulation of the gibberellin's hormonal pathway (Ji et al., 2023). These interfaces are essential for ASK1 binding and DELLA substrate recognition, however, their hydrophobicity complicates protein expression and purification because individual proteins have a high tendency to aggregate (Islam et al., 2025). Thus, it remains challenging to express SLY1 without partners or solubility tags both in prokaryotic or eukaryotic systems (Li et al., 2017; Yoshida et al., 2011), consequently limiting studies of the SLY1–DELLA interaction. Here, we examined the properties of the SLY1 structure to understand the challenges of purification and propose an efficient method to cost‐efficiently express and purify soluble SLY1 in complex with ASK1 in a bacterial system.

## 2 RESULTS AND DISCUSSION

### 2.1 Structural determinants of SLY1 hydrophobicity

**FIGURE 1:**
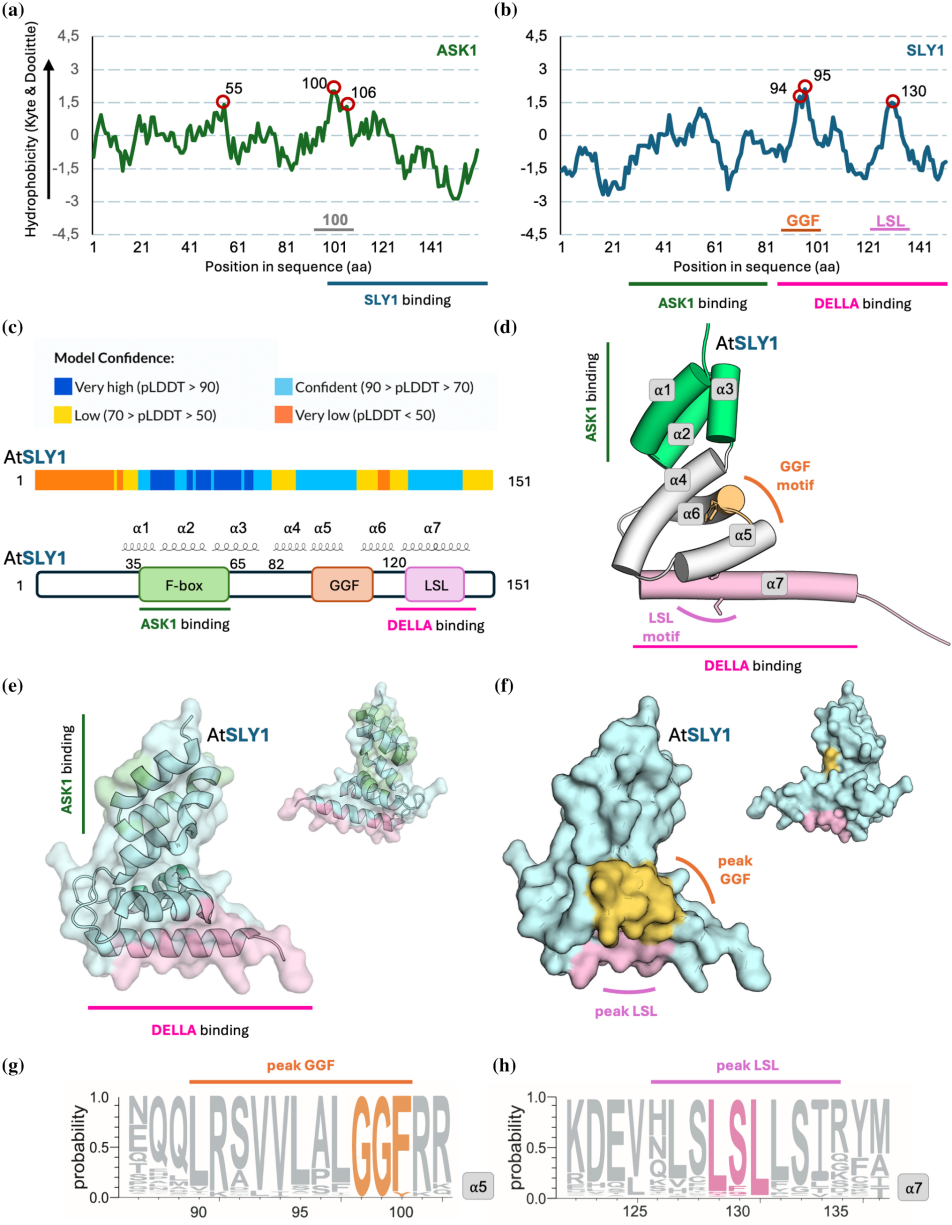
Hydrophobicity profiles and structural organization of SLY1. (a, b) Hydrophobicity profile of (a) ASK1 or (b) SLY1. In both cases, hydrophobicity scores were plotted along the amino acid sequence. Residues with scores above 0 are considered hydrophobic; those below 0 are hydrophilic. Major hydrophobic peaks are highlighted by red circles. The hydrophobic peaks are shown at the bottom of the plot. Regions involved in protein–protein interactions are indicated below each graph. (c) Top: AtSLY1 is represented by rectangles, colored according to their per‐residue pLDDT values. Bottom: Schematic representation of AtSLY1. Motifs are illustrated by boxes. Predicted AlphaFold helices are represented on top of the schematic representation. (d) Canonical structure of SLY1 from plants. The α‐helices are represented as cylinders, the N‐terminus region that contacts ASK1 is colored in green. The C‐terminus region that contacts DELLA is colored in pale blue. The position of the GGF and LSL motifs their lateral chains are depicted as sticks. Residues at the N and C‐terminus are predicted to be disordered and were omitted for clarity. (e, f) The three‐dimensional structure of AtSLY1 is represented in cartoon and surface representations. (e) Interaction surfaces are depicted in color: green for the region interacting with ASK1, pink for interaction with DELLA, and blue for the rest of the structure. (f) The position of the two major hydrophobic peaks identified by the Kyte‐Doolittle hydrophobicity plots are highlighted on the same structure: surface with the GGF motif colored in orange and the LSL motif colored in magenta, indicating potential interaction interfaces. (g, h) Web logos highlighting conserved motifs in SLY1 orthologues. (g) Web logo centered on the AtSLY1 GGF motif (orange), (h) on the LSL motif (magenta). Hydrophobic peaks identified using Kyte‐Doolittle hydrophobicity plots are highlighted respectively in orange on helix ⍺5 and pink on helix ⍺7.

The reported difficulties in expressing SLY1 (Dahal et al., 2025; Islam et al., 2025; Li et al., 2017) prompted us to investigate the structural features and critical hydrophobic residues/surfaces of SLY1. Kyte‐Doolittle hydrophobicity profiles of AtASK1 (Figure 1a) indicate that it is predominantly hydrophilic (mean KD: −0.331), with three hydrophobic peaks at V55 (+1.40), L101 (+2.01), and N106 (+1.24). Residues L101 and N106 are in a region hereafter called “peak 100,” that interacts with the F‐box domain of SLY1 (Figure 1a). In contrast, despite being predominantly hydrophilic (mean KD: −0.603), SLY1 exhibits a hydrophilic N‐terminal F‐box domain (aa 1–81) while its C‐terminal region (aa 82–151) contains hydrophobic peaks at V94 (+1.77), V95 (+2.11), and S130 (+1.5) (Figure 1b). These peaks lie near the GGF and LSL motifs, characterized as essential for DELLA interactions (Gomi et al., 2004). For the remainder of the present study, V94 and V95 together form the “GGF peak” while S130 forms the “LSL peak.” To determine the localization of these peaks in the three‐dimensional structures, AlphaFold v2 models were generated for AtSLY1 (Figure 1c,d), AtSNE1, and crop orthologs (Figure S1, Supporting Information). High pLDDT scores indicate strong confidence level. These proteins share together a common organization, composed of seven α‐helices: α1 (35–45), α2 (46–58), α3 (59–68), α4 (69–79), α5 (85–98), α6 (99–115), and α7 (121–141), with disordered extremities (aa 1–34 and 142–151) (Figure 1d). Helices α1–α3 form the conserved F‐box domain mediating ASK1 interaction, with pLDDT values >90%. Helix α4 contacts the F‐box domain, while α5 and α6, separated by the GGF motif, pack against α4 through hydrophobic interactions, stabilizing α7, which contributes to DELLA recognition via the LSL motif (Figure 1d). These models are structurally similar to the cryo‐EM structure of AtSLY1, highlighting the robustness of the AlphaFold predictions. We used AlphaFold predicted structures of SLY1 and ASK1 to map the hydrophobicity peaks identified through Kyte and Doolittle analyses on each protein surface (Figures S2, S3). Although the overall surface of the AtSLY1 F‐box domain is predominantly hydrophobic (Figure S2A), additional hydrophobic patches are observed notably along α‐helix 7, and these are conserved in SLY1 ortholog (Figure S3). ASK1 contacts helices α1–3 of SLY1 via the exposed C‐terminal “peak 100” (Figure S4). Even in complex with ASK1, ~90% of the SLY1 surface remains exposed to the solvent (Figure 1e,f), leaving substantial hydrophobic regions accessible and potentially compromising stability. Notably, the GGF and LSL peaks form an extended hydrophobic surface that interacts with DELLA (Figure 1e,f). Sequence alignment of SLY1 orthologues highlights the high conservation of these hydrophobic regions across SLY1 proteins of major cultivated plants (Figure 1g,h).

### 2.2 Expression challenges highlight the capacity of ASK1 and SLY1 to form oligomers

Given the known difficulties in expressing and purifying F‐box domain proteins (Li et al., 2017), we developed strategies for heterologous expression of SLY1 in *Escherichia coli*. Previous insect cell studies highlighted the importance of co‐expressing the F‐box protein with ASK1 to stabilize the domain (Li et al., 2017). However, the use of eukaryotic expression systems substantially increases experimental cost and complexity and introduces the risk of unwanted post‐translational modifications that could confound downstream analyses. As a first strategy developed in our laboratory and others (Correddu et al., 2019; Ganjave et al., 2026; LaVallie et al., 1993), SLY1 was co‐expressed with ASK1 in a bacterial system using the pRSF_Trx‐ASK1/SLY1 construct (Figure S5A). In this construct, ASK1 is fused to an N‐terminal Thioredoxin (Trx) tag to enhance solubility and proper folding of the protein complex, while reducing aggregation of recombinant proteins expressed in *E. coli* (Costa et al., 2014; LaVallie et al., 1993; LaVallie et al., 2000). A TEV protease cleavage site located downstream allows the release of the native ASK1/SLY1 complex from the His‐Trx tag. The Trx‐ASK1/SLY1 fusion protein expressed well in *E. coli* (Figure S5C) and, after affinity chromatography, the soluble complex was purified by size‐exclusion chromatography (Figure S5B). SDS‐PAGE analyses revealed that both ASK1 and SLY1 started eluting at the void volume, suggesting either aggregation or formation of high molecular weight complexes (fractions 4–9), whereas excess ASK1 eluted at fractions 12–14 (Figure S5C). We also designed a construct in which both proteins are fused and separated by a glycine‐serine linker (Figure S6A). This 34 kDa construct was fused to a removable 11 kDa His‐Trx cassette to enhance solubility of the complex. The fusion protein was expressed in *E. coli* and purified by affinity chromatography and gel filtration (Figure S6B). SDS‐PAGE analysis of the elution profile on a gel filtration column showed that the fusion protein eluted at an apparent molecular weight exceeding 100 kDa, substantially larger than the expected molecular mass of 44 kDa, suggesting higher‐order oligomerization (Figure S6C). TEV protease treatment yielded two populations on SDS‐PAGE: a non‐cleaved species at 44 kDa and a cleaved species migrating at the expected 34 kDa (Figure S7A) with mass spectrometry confirming that the 44‐kDa species corresponds to the non‐cleaved His‐Trx‐ASK1‐SLY1 protein fusion (Figure S7B). The incomplete cleavage likely reflects limited accessibility of the TEV site, potentially due to aggregation or oligomer formation. Expression and purification of the ASK1‐SLY1 fusion protein highlight its propensity for oligomerization (Figure S8). Although no dimer of AtASK1 or AtSLY1 has been reported, the ortholog DdSKPA1 forms stable dimers at high concentrations in vitro, as demonstrated by analytical ultracentrifugation and NMR experiments (Kim et al., 2020). Sequence and structural alignments between DdSKPA1 and AtASK1 reveal strong similarity and residue conservation (Figure S9). Collectively, the high‐molecular‐weight elution peak and partial cleavage are consistent with the formation of soluble oligomers.

### 2.3 Structural basis and possible consequences of oligomerization

**FIGURE 2:**
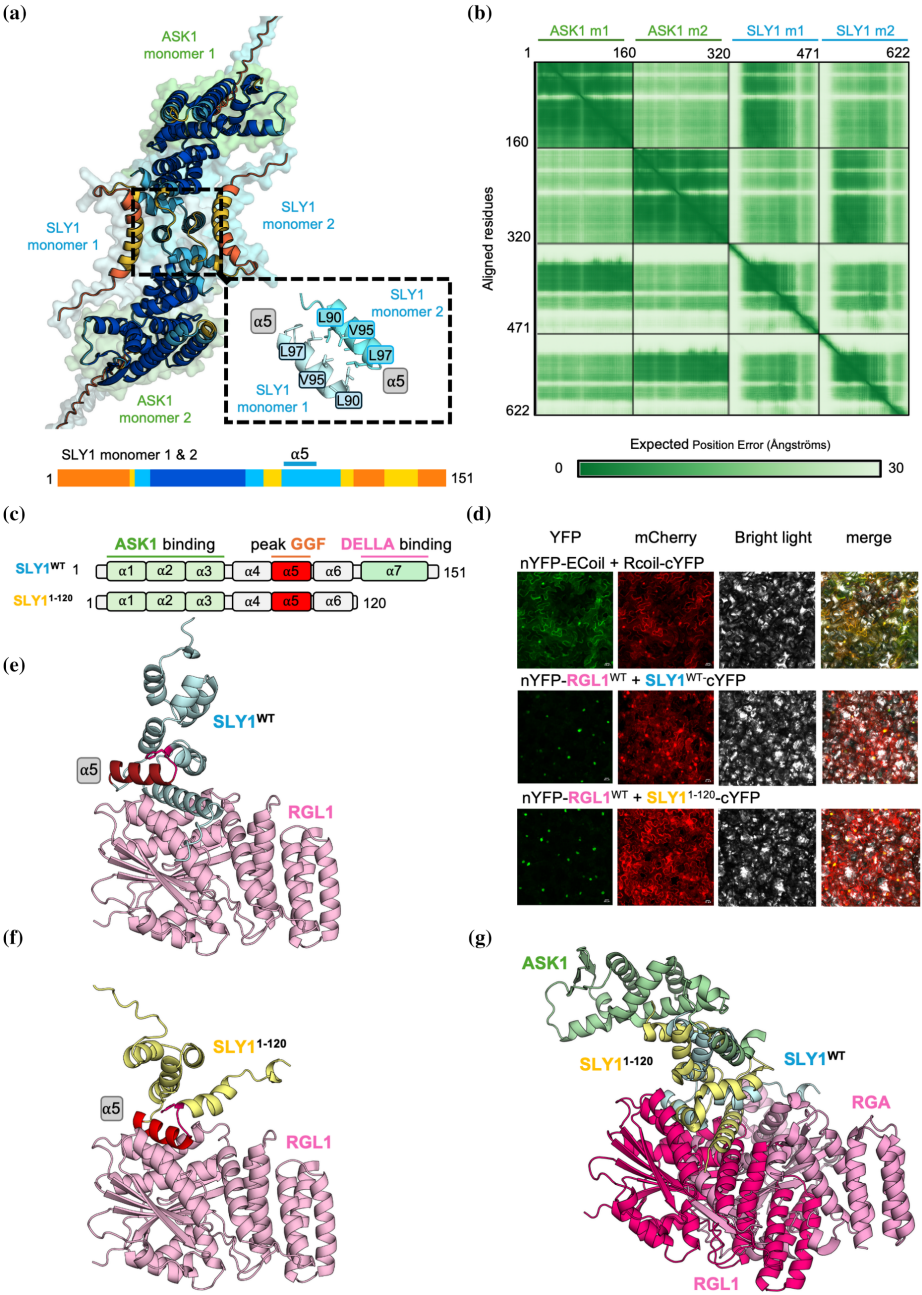
Structural model of SLY1 and functional interaction with DELLA. (a) Predicted structure of an AtASK1‐AtSLY1 complex dimer. (b) Predicted aligned error plot (PAE) for the structure presented in (a). (c) Schematic representation of SLY1^1‐151^ and truncated SLY1^1‐120^, where helices are illustrated by boxes. (d) Bimolecular fluorescence complementation assay between SLY1 and RGL1 in *Nicotiana benthamiana* leaves. In this assay nYFP‐RGL1^WT^ was co‐expressed with SLY1^1‐151^‐cYFP or SLY1^1‐120^‐cYFP. Co‐expression of nYFP‐ECoil and RCoil‐cYFP was used as a positive control. (e, f) AlphaFold predicted structure comparison of RGL1 in complex with SLY1^1‐151^ (e) or SLY1^1‐120^ (f). (g) Alignment of AlphaFold predicted structure of SLY1^1‐120^/RGL1 and cryo‐EM structure of ASK1‐SLY1‐RGA‐GID1A complex (PDB: 9O4K).

AlphaFold predictions reveal the possible formation of ASK1 dimers (Figure S10), in agreement with previous NMR structural analyses showing that dimerization could occur through the hydrophobic peak 100 on helix α5 of DdSKPA1 (Figure S11). Such dimerization may represent a regulatory mechanism for SCF activity, whereby burial of the peak 100 hydrophobic surface partially masks ASK1 interaction sites and limits association with the F‐box domain (Kim et al., 2020). An ancestral version of SLY1 from *M. polymorpha* exhibits a more flexible C‐terminus capable of adopting multiple orientations, allowing interactions with various DELLA and GRAS proteins (Ji et al., 2023). In AlphaFold predictions of SLY1 without partners, individual α‐helices displayed pLDDT values exceeding 70%, whereas modeling in the presence of DELLA increased confidence in predicted secondary structures to over 90% (Figure S12). Comparison of predicted AtSLY1 with the experimentally determined AtSLY1 structure in complex with AtASK1 and AtRGA (PDB 9O4K) revealed structural compaction, likely reflecting increased rigidity of helix α7. Indeed, this helix is more flexible and partially detached in the absence of DELLA (Figure S12). AlphaFold predictions of MpSLY1 dimers reveal a dimeric organization centered on helix α5, with hydrophobic surfaces buried at the interface to stabilize the interaction (Figure S13). Structural modeling of AtSLY1 dimers in complex with ASK1 highlights a possible interface between two SLY1 monomers (Figures 2a,b and S14) and the potential role of helix α5 in dimer formation. The α5 helix of each monomer contains the hydrophobic GGF surface capable of forming interactions with L90, L97, V94, and V95. To assess whether loss of helix α7 abolishes DELLA binding, we performed bimolecular fluorescence complementation experiments in *Nicotiana benthamiana* leaves. Truncated SLY1 (1–120), lacking helix α7 and exposing α5 at the C‐terminus, remains functional and interacts with DELLA in the nucleus (Figure 2c,d). The AlphaFold model of a DELLA‐SLY1^1‐120^ complex indicated that helix α5 can occupy the same position as helix α7 in the hydrophobic groove of DELLA, suggesting that exposure of helix α5 compensates for the lack of helix α7 (Figure 2e,f). This phenotype has been associated with dwarfism (Gomi et al., 2004), which may arise from altered spatial organization of the SCF^SLY1^ complex, potentially affecting substrate positioning relative to the E2‐ubiquitin conjugating enzyme (Figures 2g and S15). In the bryophyte *M. polymorpha*, where gibberellin‐mediated regulation is absent (Hernández‐García et al., 2019; Ji et al., 2023), SLY1 homologs are thought to operate in a deferent regulatory context. These observations suggest that α5‐centered dimerization may represent an ancestral autoregulatory mechanism. Such an arrangement would shield the DELLA‐interacting surface, providing a potential dimerization‐based mechanism for modulating growth through reduced DELLA degradation. Dimerization centered around helix α5 may represent a molecular remnant of an ancestral autoregulatory system.

### 2.4 Soluble expression of ASK1/SLY1 through association with a maltose binding protein

**FIGURE 3:**
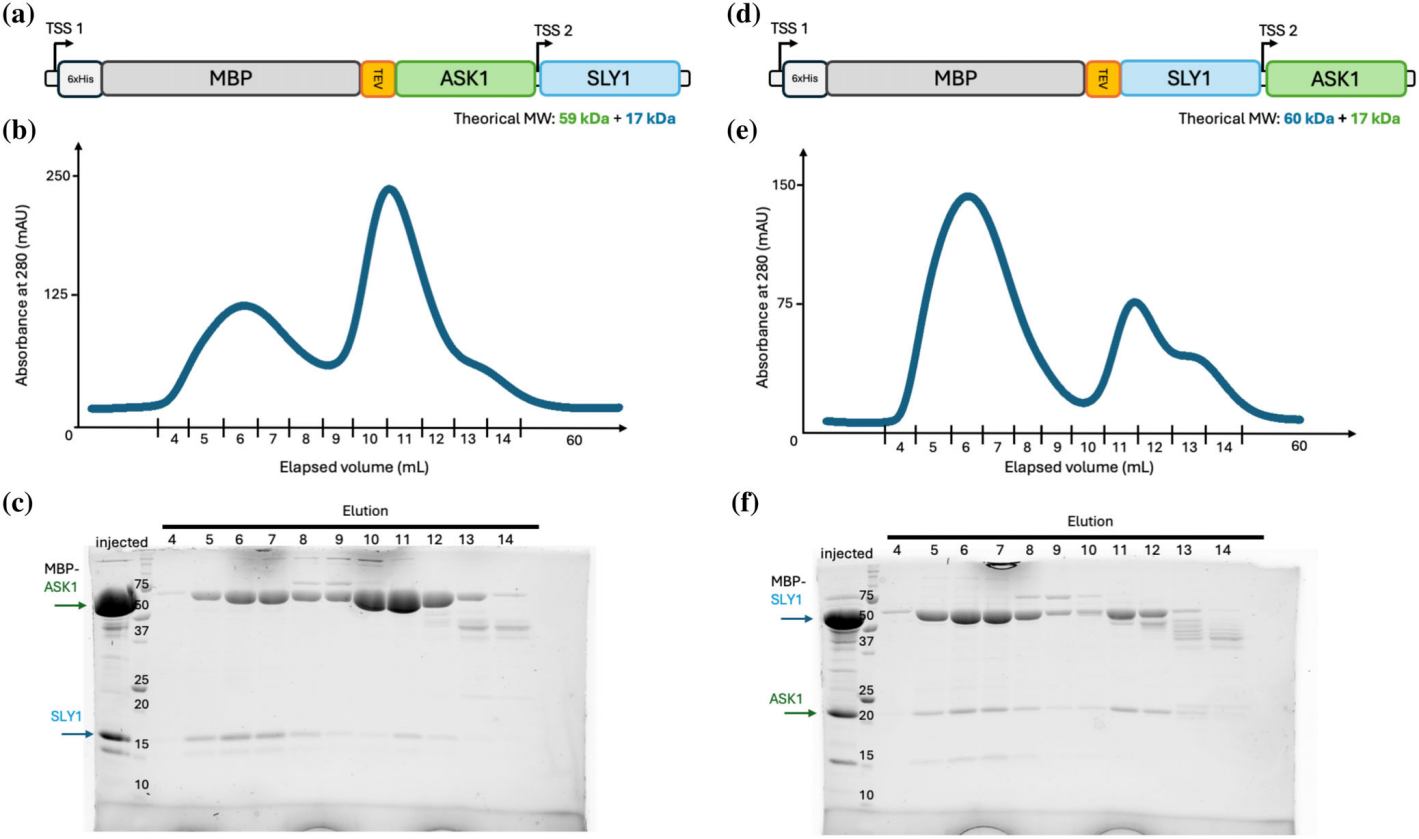
The MBP tag improves solubility of ASK1/SLY1 complexes. (a) Schematic representation of the strategy used for co‐expressing MBP‐ASK1 and SLY1 in *Escherichia coli*. Transcription start sites (TSS) are represented in front of the expressed proteins. Motifs and proteins are depicted by colored rectangles: 6xHis tag for purification in pale gray, solubilizing maltose‐binding protein (MBP) tag in gray, tobacco each virus cleavage motif in orange, ASK1 in green and SLY1 in blue. (b) Gel‐filtration chromatogram of co‐expressed MBP‐SLY1/ASK1. (c) SDS‐PAGE analysis of gel‐filtration fractions shown in (B). (d) Schematic representation of the strategy used for co‐expressing MBP‐SLY1 and ASK1 in *E. coli*. Transcription start sites (TSS) are represented in front of the expressed proteins. Motifs and proteins are depicted by colored rectangles: 6xHis tag for purification in pale gray, solubilizing maltose‐binding protein (MBP) tag in gray, tobacco each virus cleavage motif in orange, SLY1 in blue and ASK1 in green. (e) Gel‐filtration chromatogram of co‐expressed MBP‐ASK1/SLY1. (f) SDS‐PAGE analysis of gel‐filtration fractions shown in (e). In (c) and (f), a band at ~75 kDa corresponds to the endogenous *E. coli* HSP70 chaperone DnaK. The presence of this protein is also consistent with the exposure of hydrophobic residues.

To compensate for the hydrophobic nature of the SLY1 C‐terminus, we co‐expressed ASK1 to stabilize the N‐terminal region and fused a maltose binding protein (MBP) tag of the construct to further enhance the solubility of the complex. The rationale behind the choice of MBP rests on its well documented ability to enhance protein solubility by promoting proper folding and suppressing aggregation (Costa et al., 2014; Sun et al., 2010). This property is particularly relevant for SLY1, whose exposed hydrophobic regions and flexible helices are predicted to promote oligomerization and aggregation when expressed alone. We therefore designed two new constructs, pRSF‐MBP: ASK1/SLY1 (Figure 3a–c) and pRSF‐MBP: SLY1/ASK1 (Figure 3d–f), where the solubility‐enhancing MBP tag was fused to the N‐terminus of ASK1 or SLY1, placed just upstream of its complementary partner in the same vector. The constructs were expressed in *E. coli*. After a first affinity chromatography step, the soluble complexes were purified by size‐exclusion chromatography. As indicated by SDS‐PAGE analysis of elution fractions of the gel filtration column, this strategy significantly improved the solubility of SLY1 with both constructs. Indeed, the presence of both ASK1 and SLY1 is clearly observed, co‐eluting together in a single soluble fraction (Figure 3c,f). These results suggest that SLY1/ASK1 co‐expression, combined with the use of a MBP tag on either partner, allows efficient expression of soluble SLY1 in a bacterial system. Overall, our results demonstrate that the interplay between hydrophobic surface exposure, helix flexibility, and potential dimerization interfaces fundamentally governs SLY1 instability in vitro, explaining its strict dependence on partners and solubility tags for successful heterologous expression and purification.

## 3 MATERIALS AND METHODS

### 3.1 Sequence alignments and hydrophobicity analyses

ASK1 and SLY1 protein sequences from *A. thaliana* and other major crops were obtained from UniProtKB, and accession numbers are listed in Table S1. Additional SLY1 sequences, including non‐crop plants, were obtained from HMMER v3.4 (Finn et al., 2011) (Table S2). Sequences were aligned using NCBI BLAST (Altschul & Lipman, 1990) and visualized with WebLogo 3 (Crooks et al., 2004). Hydrophobicity profiles of AtASK1, AtSLY1, AtDELLAs, and crop SLY1 proteins were analyzed using ProtScale (ExPASy) with the Kyte & Doolittle algorithm (Gasteiger et al., 2005) and a sliding 9‐residue window.

### 3.2 Structural prediction of SLY1 complexes

The structure of isolated AtSLY1, AtSNE1, SLY1s, and AtSLY1 dimer and complexes composed of AtSLY1/AtASK1 and AtSLY1/AtDELLAs were obtained through structure prediction using AlphaFold v2.0 or 3.0, as indicated. The visualization software PyMOL (The PyMOL Molecular Graphics System, Version 2.5.2 Schrödinger, LLC.) was used to analyze the structures, perform structural alignments, and generate figures.

### 3.3 Molecular cloning

To facilitate purification and functional studies, all constructs possess an N‐terminal hexa‐histidine tag (His‐tag) with either a thioredoxin (Trx) or a maltose‐binding protein (MBP), as well as a tobacco etch virus (TEV) protease recognition site (ENLYFQGS) to facilitate the separation between the tags and the target proteins. ASK1^1‐160^ and SLY1^1‐151^ full‐length sequences were obtained by polymerase chain reaction (PCR) amplification from an *Arabidopsis* cDNA library. The corresponding ASK1 and SLY1 sequences were then cloned by GIBSON assembly into either BamHI (MCS1) or NdeI (MCS2) sites of homemade pRSF‐Trx or pRSF‐MBP Duet vectors using oligonucleotides listed in Table S3, generating the following constructs: pRSF_MBP‐SLY1^1‐151^, pRSF_Trx‐ASK1^1‐160^‐SLY1^1‐151^, pRSF_MBP‐ASK1^1‐160^‐SLY1^1‐151^, and pRSF_MBP‐SLY1^1‐151^‐ASK1^1‐160^. Full‐length ASK1 and SLY1 were also cloned as linear fusion proteins into the BamHI site of homemade pRSF‐Trx vector to generate pRSF_Trx‐ASK1‐SLY1^Fus^ where both proteins are part of a single polypeptide separated by a GS linker. The integrity of all constructs was confirmed by Sanger sequencing (Génome Québec) or Nanopore sequencing (Plasmidsaurus). Constructs were transformed into *E. coli* strain BL21(DE3) CodonPlus RIL prior to protein expression.

### 3.4 Heterologous protein expression in *E. coli*

Transformed *E. coli* BL21(DE3) CodonPlus pRIL were grown overnight at 37°C in LB medium (10 g/L tryptone, 5 g/L yeast extract and 10 g/L NaCl) containing the appropriate antibiotics (34 μg/mL chloramphenicol, 50 μg/mL kanamycin). Precultures were diluted with chilled Super Broth medium (32 g/L of tryptone, 20 g/L of yeast extract and 5 g/L of NaCl) containing antibiotics and 300 μM isopropyl‐β‐D‐thiogalactopyranoside (IPTG). Protein expression was performed at 18°C for 24 h. Cells were pelleted by centrifugation at 10000 ×g for 40 min and resuspended in lysis buffer (20 mM Tris–HCl, pH 8.0, 500 mM NaCl, 20 mM imidazole pH 8.0, 5 mM β‐mercaptoethanol and 0.5 mg/mL of lysozyme). Cells were lysed by sonication (Branson Sonifier 450) for 3 cycles of 2 min, at power of 200 W and a 1:1 duty cycle, then centrifugated at 48500 g for 30 min. The supernatant was applied to a Ni‐NTA resin (QIAGEN). Proteins were eluted with 20 mM Tris–HCl, pH 8.0, 500 mM NaCl, 500 mM imidazole pH 8.0, 5 mM β‐mercaptoethanol. All proteins were purified by gel‐filtration chromatography (Superdex 200 pg. 10/300, Cytiva) in gel filtration buffer composed of 20 mM Tris–HCl, pH 8.0, 150 mM NaCl, 5 mM β‐mercaptoethanol. Fractions were analyzed by SDS‐PAGE (15% polyacrylamide gels).

### 3.5 Plant material and growth conditions

*Nicotiana benthamiana* (PI 555478 USDA) were grown in a mixture of black earth, perlite and peat moss (2:1:1, v:v:v) in E15 Conviron growth cabinet at 22°C and 70% relative humidity, under a photon flux density of 100 μmol m^−2^ s^−1^ (fluorescent and incandescent lighting) with a 16 h/8 h (day/night) photoperiod. Plants were watered with 20:20:20 N:P:K at a concentration of 0.5 g/L, and infiltration was performed on 5‐week‐old leaves.

### 3.6 Bimolecular complementation by fluorescence assay

To assess SLY1‐RGL1 interactions, we amplified the coding sequences of SLY1 and RGL1 and inserted these sequences between the XbaI and NcoI restriction sites of a modified pAVA321 vector (Von Arnim et al., 1998) encoding 35S:nYFP^1‐156^ or pAVA 35S:cYFP^155‐240^ vectors (Jmii et al., 2025). RGL1^WT^, SLY1^1‐151^, SLY1^1‐120^ sequences were cloned into pAVA modified vectors using Gibson assembly using the XbaI site, to generate pAVA 35S:RGL1^WT^‐nYFP, pAVA 35S:SLY1^WT^‐cYFP, and pAVA 35S:SLY1^1–120^‐cYFP. The resulting cassettes were excised and inserted in the pPZP vector in the SmaI and KpnI sites to generate pPZP 2X35S:RGL1^WT^‐nYFP, pPZP 2X35S:SLY1^WT^‐cYFP, and pPZP 2X35S:SLY1^1‐120^‐cYFP. We used pPZP 2X35S:ECoil‐cYFP, pPZP 2X35S:RCoil‐nYFP as a positive control (Doh et al., 2018), whereas pPZP 2X35S:mCherry signal served as a marker for transient protein expression and localization of agro‐infiltrated tissue in *N. benthamiana* leaves. All pPZP plasmids mentioned previously were transformed into *Agrobacterium tumefaciens* EHA105 to generate individual strains. Agroinfiltrations of *N. benthamiana* leaves were performed as described (Bouard et al., 2024). Two days after infiltration, fluorescence excitation/emission wavelengths of 488 nm/525 nm for eYFP and 561 nm/595 nm for mCherry were used on a Nikon A1+ confocal laser scanning microscope. Images were analyzed using Fiji software (Schindelin et al., 2012).

## AUTHOR CONTRIBUTIONS

Souleïmen Jmii and Laurent Cappadocia designed the experiments. Souleïmen Jmii, William Bouard, Mathilde Rochas collected data. Souleïmen Jmii prepared all the figures and tables. Souleïmen Jmii drafted the manuscript. Souleïmen Jmii, William Bouard, François Dragon and Laurent Cappadocia edited the manuscript and approved the submitted version.

## CONFLICT OF INTEREST STATEMENT

The authors declare that the research was conducted in the absence of any commercial or financial relationships that could be construed as a potential conflict of interest.

## Supporting information


**Figure S1.** AlphaFold‐predicted structures of SLY1 from different crops species. Three‐dimensional structures of SLY1 proteins were generated by AlphaFold v.2 and depicted in cartoon representation. The N‐ and C‐terminal regions, predicted with low confidence, were truncated for clarity in the structural representations. The figure highlights the seven α‐helices forming the core of the structure. (A) AtSLY1. (B) AtSNE1. (C) OsSLY1 from rice. (D) GmSLY1 from soybean. (E) ZmSLY1 form maize. (F) BnSLY1 from canola. Proteins are colored according to their per‐residue pLDDT values.
**Figure S2.** AlphaFold‐predicted structures of SLY1 and ASK1. (A) SLY1 and (B) ASK1 are depicted in surface representation. Electrostatic surface potentials were calculated using APBS and mapped onto the molecular surface. Blue indicates positive electrostatic potential, red indicates negative electrostatic potential, and white indicates near‐neutral potential.
**Figure S3.** AlphaFold‐predicted structures of SLY1s. SLY1 proteins are depicted in surface representation. Electrostatic surface potentials were calculated using APBS and mapped onto the molecular surface. Blue indicates positive electrostatic potential, red indicates negative electrostatic potential, and white indicates near‐neutral potential. (A) AtSLY1 (B) AtSNE1 (C) BnSLY1 from Brassica napus (canola) (D) OsSLY1 from Oryzia sativa (rice). (E) GmSLY1 from Glycine max (soybean). (F) ZmSLY1 from Zea maize (maize).
**Figure S4.** Structure of ASK1. The three‐dimensional structure of AtASK1 from cryo‐EM structure (PDB: 9O4K) is shown in cartoon and surface representations. (A) The interaction surfaces for SLY1 are depicted in teal. (B) The “Peak 100” hydrophobic peak identified by the Kyte‐Doolittle hydrophobicity scale is highlighted on the structure (gray) and corresponds to α‐helix 5 of ASK1.
**Figure S5.** Purification of co‐expressed ASK1/SLY1 by gel filtration chromatography. (A) Schematic representation of the strategy used for co‐expressing ASK1 and SLY1 in *Escherichia coli*. Transcription starts sites (TSS) are represented in front of the expressed proteins. Motifs and proteins are depicted by colored rectangles: 6× Histidine tag for purification in pale gray, thioredoxin solubilizing tag (Trx) in gray, tobacco each virus cleavage motif (ENLYFQGS) in yellow, ASK1 (green) and SLY1 (blue). (B) Chromatogram from gel filtration chromatography of the co‐expressed ASK1/SLY1. (C) SDS PAGE corresponding to the gel filtration chromatography of the co‐expressed ASK1‐SLY1. A band at ~75 kDa corresponds to the endogenous *E. coli* HSP70 chaperone, which we frequently observe as a co‐purifying contaminant under our purification conditions.
**Figure S6.** Purification of linear fusion of ASK1‐SLY1 by gel filtration chromatography. (A) Schematic representation of the strategy used for co‐expressing ASK1 and SLY1 in *Escherichia coli*. Transcription start site (ATG) are represented in front of the expressed protein. Motifs and proteins are depicted by colored rectangles: 6× Histidine tag for purification in pale gray, thioredoxin solubilizing tag (Trx) in gray, tobacco each virus cleavage motif (ENLYFQGS) in yellow, and the linear fused proteins ASK1‐SLY1 in green. (B) Chromatogram from gel filtration chromatography of the linear fusion of ASK1‐SLY1. (C) SDS‐PAGE corresponding to the gel filtration chromatography of linear fusion of ASK1‐SLY1. A band at ~75 kDa corresponds to the endogenous *E. coli* HSP70 chaperone, which we frequently observe as a co‐purifying contaminant under our purification conditions.
**Figure S7.** Mass spectroscopy analysis of the ASK1‐SLY1 fusion. (A) SDS‐PAGE analysis of samples obtained by gel‐filtration purification of TEV‐cleaved linear fusion of ASK1‐SLY1. Black box: Purified linear fusion Trx‐ASK1‐SLY1 identified by LC–MS/MS. Green box: ASK1‐SLY1 generated after TEV cleavage. Yellow box: TEV protease. Gray box: Thioredoxin solubility tag. (B) Non cleaved protein from black box was analyzed by LC–MS/MS confirming the amino acid sequence and identity of the protein of the linear fusion of ASK1‐SLY1.
**Figure S8.** Schematic model of potential oligomeric superstructures formed by fused ASK1‐SLY1. Three distinct interaction interfaces are depicted: (1) Cross‐interaction between ASK1 and SLY1. (2) ASK1 homodimer. (3) SLY1 homodimer. Proposed oligomeric architectures are based on structural and biochemical evidence.
**Figure S9.** Alignment of DdSKPA1 structures. (A) Alignment of three‐dimensional structures of monomeric SKPA1 from Dictyostelium discoideum, solved by NMR (light blue, PDB: 6V88), predicted by AlphaFold (forest green), and solved by cryo‐EM (light green, PDB: 9O4K). (B) Alignment of dimeric structures: the NMR structure of DdASKPA1 (turquoise, residues 1–116) and the AlphaFold prediction of AtASK1 (gray, residues 1–127). (C) Sequence alignment of DdSKP1 and AtASK1.
**Figure S10.** Predicted dimerization of AtASK1. (A) Predicted three‐dimensional structure of AtASK1. The protein is shown in light green on cartoon and surface representations, and the hydrophobic patch predicted by the Kyte‐Doolittle scale is colored in gray on helix α5 and labeled “dimerization patch.” (B) Predicted three‐dimensional structure of an AtASK1 dimer complex. Monomer 2 is shown in dark green, and the hydrophobic patch is highlighted in gray on the second α5 helix. (C) The predicted aligned error (PAE) diagram obtained with AlphaFold v3 suggests an interaction between two monomers of AtASK1.
**Figure S11.** Dimerization of SKPA1 from Dictyostelium discoideum. (A) NMR structure of a monomeric SKPA1 (PDB: 6V88). The protein is shown in light green, cartoon, and surface representation, while the hydrophobic patch predicted by the Kyte‐Doolittle scale is depicted in mauve on helix α5 and labeled as “dimerization patch” corresponding to the putative dimerization surface. (B) NMR structure of the DdSKPA1 dimer. The monomer is shown in teal, and the hydrophobic patch is highlighted in mauve on the second α5 helix.
**Figure S12.** Structural consequence of SLY1 binding to ASK1 or DELLA. (A) pLDDT diagrams for SLY1 modeled alone or in complex with ASK1 or DELLA. SLY1 proteins are represented by rectangles, colored according to their per‐residue pLDDT values. (B) Structural alignment of AlphaFold‐predicted structures of SLY1 in apo or complexed forms along with the cryo‐EM structure depicted in yellow (PDB: 9O4K).
**Figure S13.** Predicted dimeric structures of MpSLY1. (A) Structure of a MpSLY1 dimer predicted with AlphaFold v3. (B) Predicted aligned error (PAE) diagram for the MpSLY1 dimeric structure shown in (A). The PAE diagram suggests an interaction between two monomers of AtASLY1. (C) Alignment of SLY1 from *Arabidopsis thaliana* and *Marchantia polymorpha*. The position of the α‐helices of AtSLY1 are indicated on top of the alignment.
**Figure S14.** AlphaFold‐predicted structures of AtSLY1 dimers. (A) Predicted aligned error plot (PAE) obtained by AlphaFold v3 suggests (B) an interaction between two monomers of AtASLY1.
**Figure S15.** Structural comparison of SLY11‐151‐RGL1WT and SLY11‐120‐RGL1WT complexes generated by AlphaFold. (A) AtSLY11‐151 and SLY11‐120 are represented by rectangles, colored according to their per‐residue pLDDT values. (B) AtSLY11‐151/AtRGL1WT. (C) AtSLY11‐120/RGL1WT. AlphaFold predicted interaction structure between SLY1, and DELLA are colored according to their per‐residue pLDDT values. (D) Close up view on the alignment of SLY1‐1‐151 and SLY1 1–120 interacted with DELLA.

**Table S1.** Accession numbers for ASK1, SLY1s and DELLA used in strutural modelization by AlphaFold prediction.
**Table S2.** Information on the SLY1s proteins used in alignment and Web Logo 3 visualizer.
**Table S3.** Primers used in this study.

## Data Availability

The data that support the findings of this study are available from the corresponding author upon reasonable request.
