# Supplementary material for: Structure‐guided optimization of SLY1 expression and purification in Escherichia coli
Source: Protein Sci. 2026 Apr 20;35(5):e70592. doi: 10.1002/pro.70592 (PMC13096574; doi:10.1002/pro.70592)
Supplement: Supplementary file 1 — Figure S1. AlphaFold‐predicted structures of SLY1 from different crops species. Three‐dimensional structures of SLY1 proteins were generated by AlphaFold v.2 and depicted in cartoon representation. The N‐ and C‐terminal regions, predicted with low confidence, were truncated for clarity in the structural representations. The figure highlights the seven α‐helices forming the core of the structure. (A) AtSLY1. (B) AtSNE1. (C) OsSLY1 from rice. (D) GmSLY1 from soybean. (E) ZmSLY1 form maize. (F) BnSLY1 from canola. Proteins are colored according to their per‐residue pLDDT values. Figure S2. AlphaFold‐predicted structures of SLY1 and ASK1. (A) SLY1 and (B) ASK1 are depicted in surface representation. Electrostatic surface potentials were calculated using APBS and mapped onto the molecular surface. Blue indicates positive electrostatic potential, red indicates negative electrostatic potential, and white indicates near‐neutral potential. Figure S3. AlphaFold‐predicted structures of SLY1s. SLY1 proteins are depicted in surface representation. Electrostatic surface potentials were calculated using APBS and mapped onto the molecular surface. Blue indicates positive electrostatic potential, red indicates negative electrostatic potential, and white indicates near‐neutral potential. (A) AtSLY1 (B) AtSNE1 (C) BnSLY1 from Brassica napus (canola) (D) OsSLY1 from Oryzia sativa (rice). (E) GmSLY1 from Glycine max (soybean). (F) ZmSLY1 from Zea maize (maize). Figure S4. Structure of ASK1. The three‐dimensional structure of AtASK1 from cryo‐EM structure (PDB: 9O4K) is shown in cartoon and surface representations. (A) The interaction surfaces for SLY1 are depicted in teal. (B) The “Peak 100” hydrophobic peak identified by the Kyte‐Doolittle hydrophobicity scale is highlighted on the structure (gray) and corresponds to α‐helix 5 of ASK1. Figure S5. Purification of co‐expressed ASK1/SLY1 by gel filtration chromatography. (A) Schematic representation of the strategy used for co‐expressing [file PRO-35-e70592-s001.pdf]

Supplementary Material

Structure-guided optimization of SLY1 expression and purification in *E. coli*

Souleïmen Jmii, William Bouard, Mathilde Rochas, François Dragon and Laurent Cappadocia

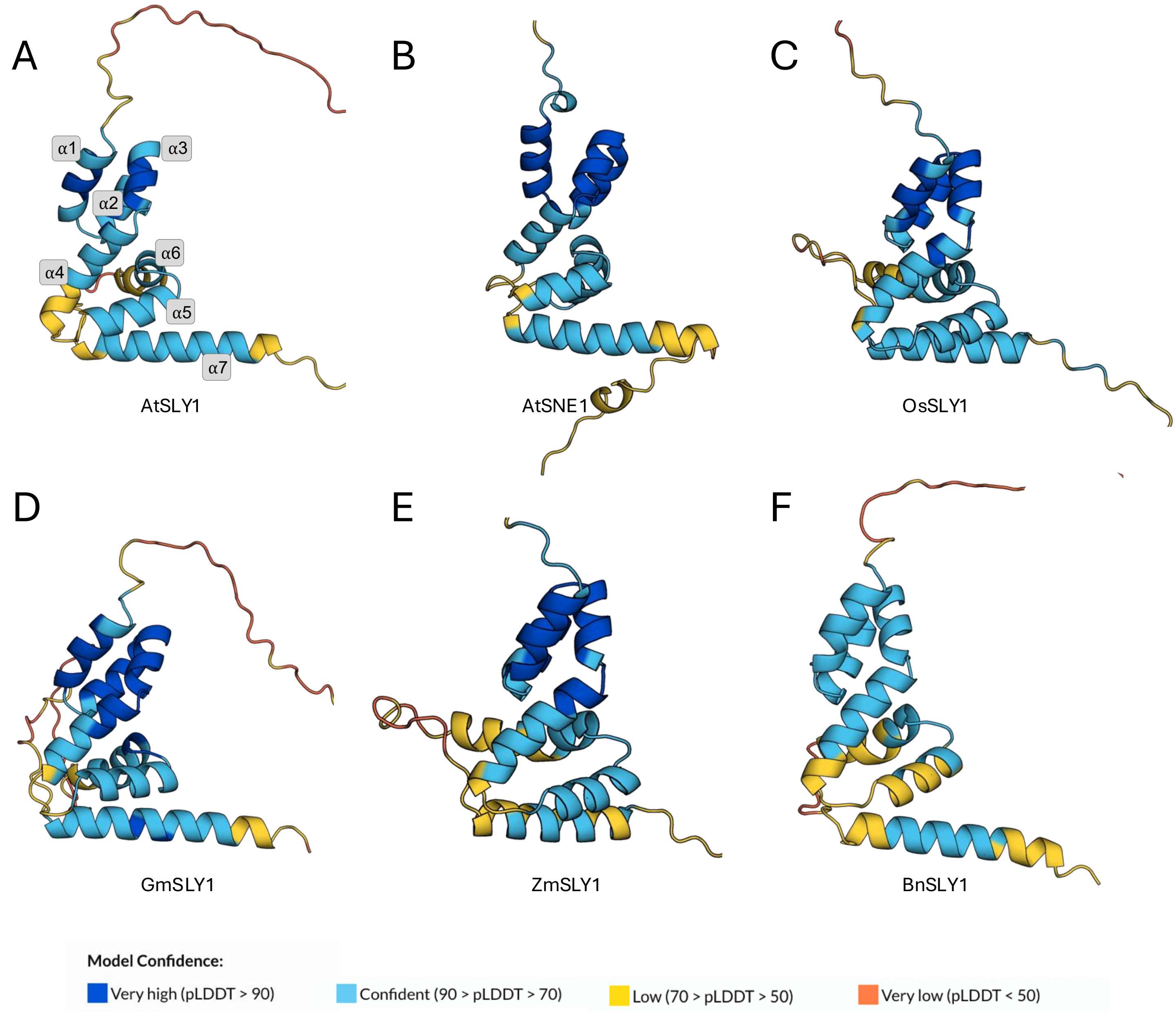

Figure S1. AlphaFold-predicted structures of SLY1 from different crops species. Three-dimensional structures of SLY1 proteins were generated by AlphaFold v.2 and depicted in cartoon representation. The N- and C-terminal regions, predicted with low confidence, were truncated for clarity in the structural representations. The figure highlights the seven α-helices forming the core of the structure. **(A)** AtSLY1. **(B)** AtSNE1. **(C)** OsSLY1 from rice. **(D)** GmSLY1 from soybean. **(E)** ZmSLY1 form maize. **(F)** BnSLY1 from canola. Proteins are colored according to their per-residue pLDDT values.

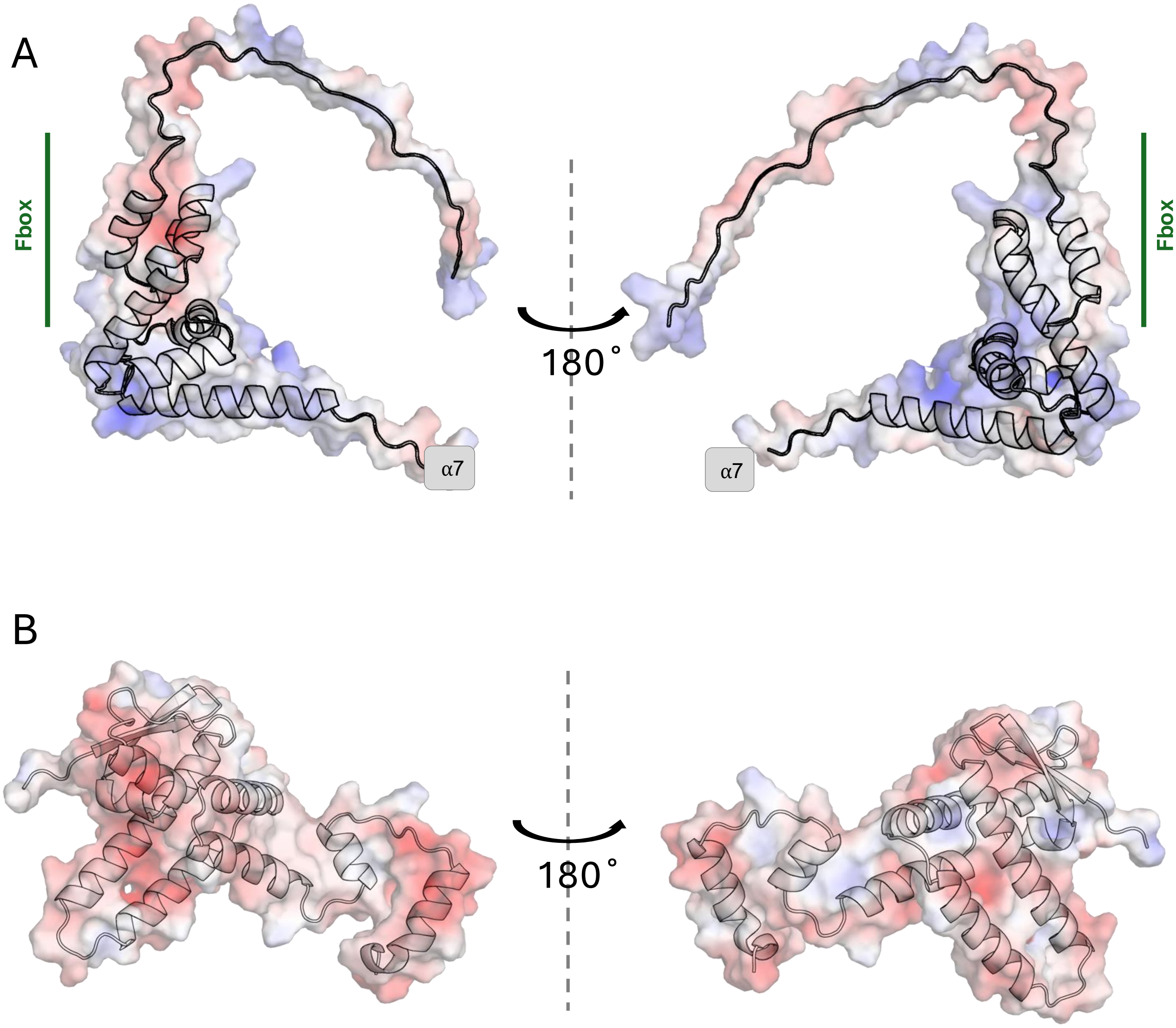

Figure S2. AlphaFold-predicted structures of SLY1 and ASK1. (A) SLY1 and **(B)** ASK1 are depicted in surface representation. Electrostatic surface potentials were calculated using APBS and mapped onto the molecular surface. Blue indicates positive electrostatic potential, red indicates negative electrostatic potential, and white indicates near-neutral potential.

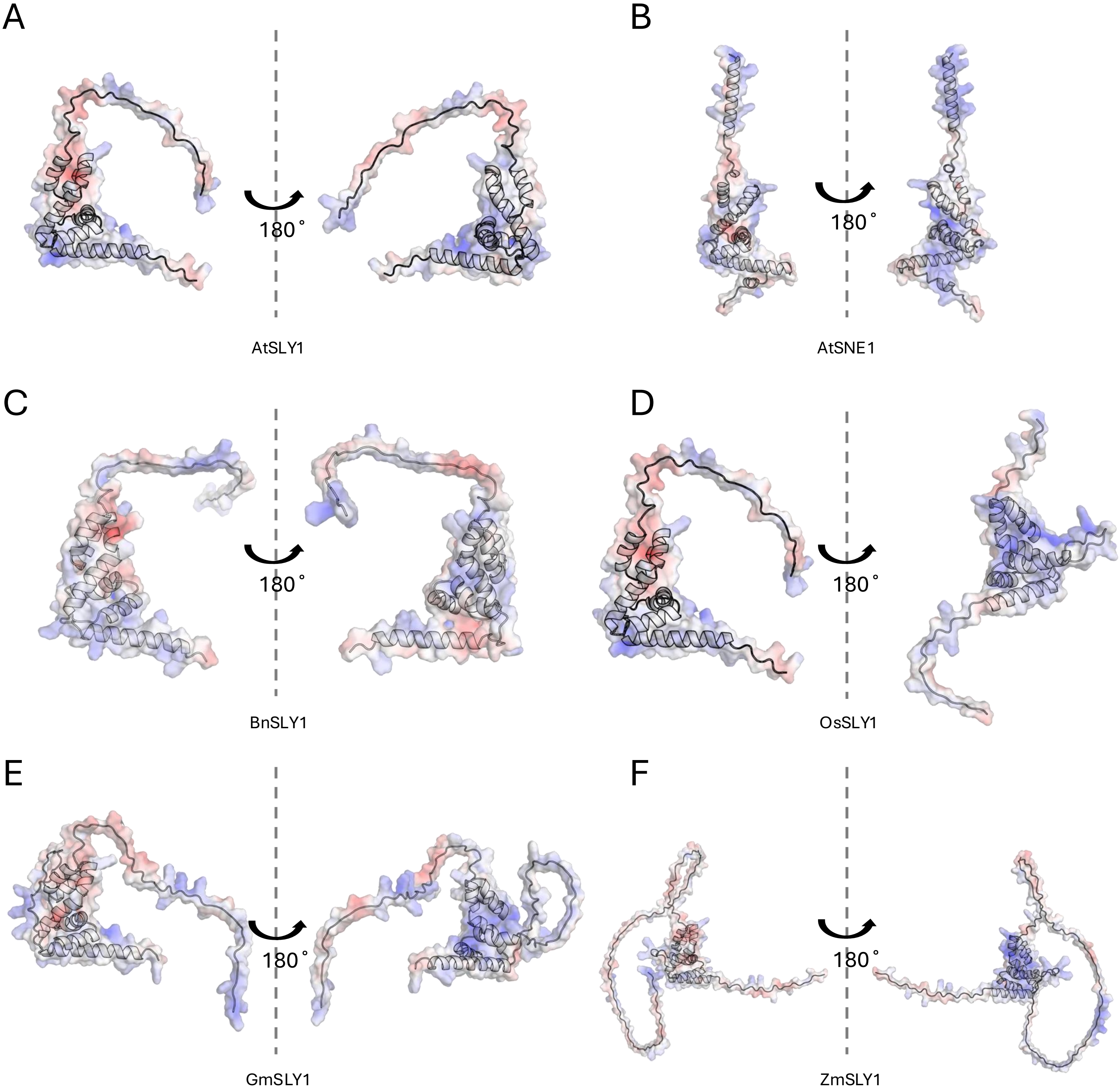

Figure S3. AlphaFold-predicted structures of SLY1s. SLY1 proteins are depicted in surface representation. Electrostatic surface potentials were calculated using APBS and mapped onto the molecular surface. Blue indicates positive electrostatic potential, red indicates negative electrostatic potential, and white indicates near-neutral potential. **(A)** AtSLY1 **(B)** AtSNE1 **(C)** BnSLY1 from *Brassica napus* (canola) **(D)** OsSLY1 from *Oryzia sativa* (rice). **(E)** GmSLY1 from *Glycine max* (soybean). **(F)** ZmSLY1 from *Zea maize* (maize).

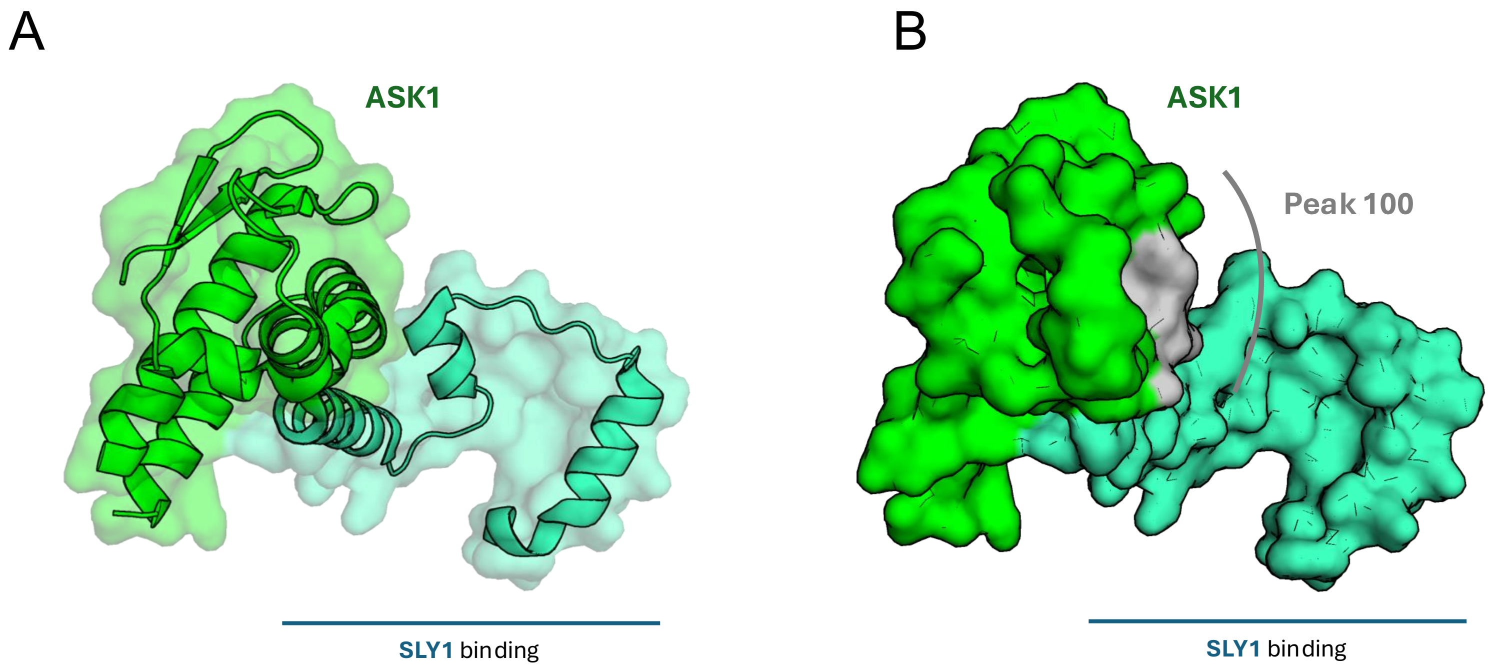

Figure S4. Structure of ASK1. The three-dimensional structure of AtASK1 from cryo-EM structure (PDB: 9O4K) is shown in cartoon and surface representations. **(A)** The interaction surfaces for SLY1 are depicted in teal. **(B)** The "Peak 100" hydrophobic peak identified by the Kyte-Doolittle hydrophobicity scale is highlighted on the structure (grey) and corresponds to α-helix 5 of ASK1.

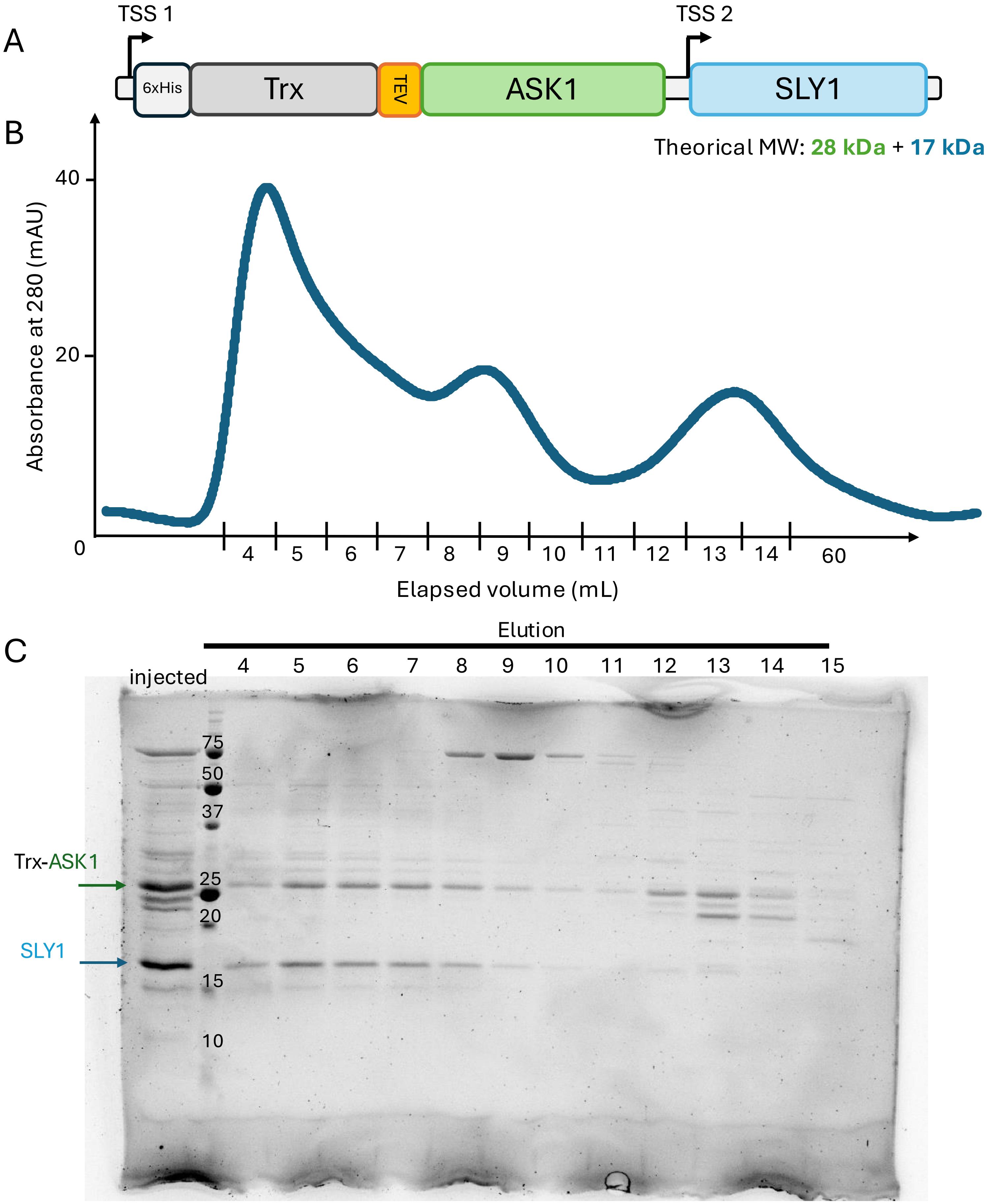

Figure S5. Purification of co-expressed ASK1/SLY1 by gel filtration chromatography. (A) Schematic representation of the strategy used for co-expressing ASK1 and SLY1 in *E. coli*. Transcription starts sites (TSS) are represented in front of the expressed proteins. Motifs and proteins are depicted by colored rectangles: 6xHistidine tag for purification in pale grey, thioredoxin solubilizing tag (Trx) in grey, tobacco each virus cleavage motif (ENLYFQGS) in yellow, ASK1 (green) and SLY1 (blue). **(B)** Chromatogram from gel filtration chromatography of the co-expressed ASK1/SLY1. **(C)** SDS PAGE corresponding to the gel filtration chromatography of the co-expressed ASK1-SLY1. A band at ~75 kDa corresponds to the endogenous E. coli HSP70 chaperone, which we frequently observe as a co-purifying contaminant under our purification conditions.

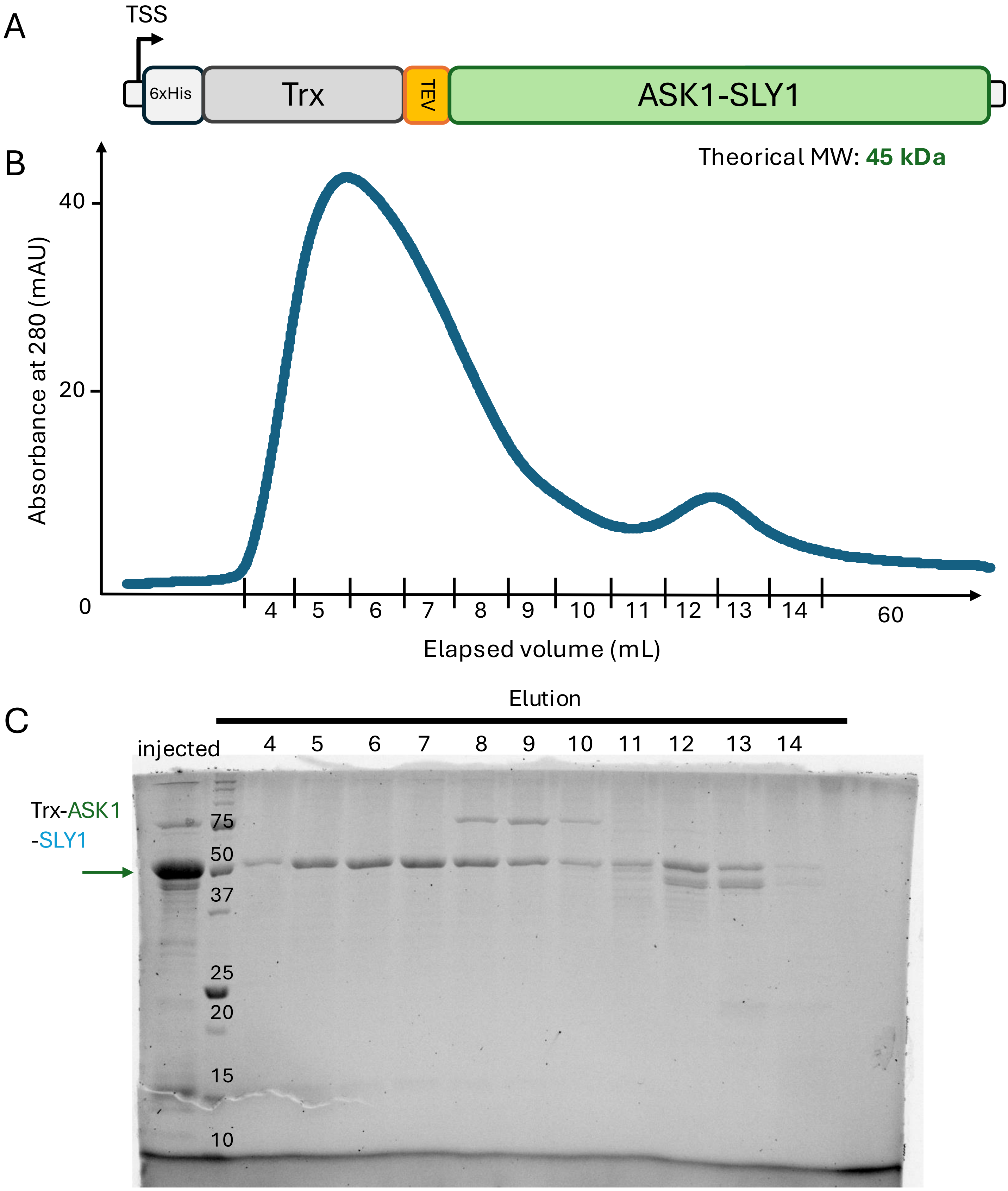

Figure S6. Purification of linear fusion of ASK1-SLY1 by gel filtration chromatography. (A) Schematic representation of the strategy used for co-expressing ASK1 and SLY1 in *E.coli*. Transcription start site (ATG) are represented in front of the expressed protein. Motifs and proteins are depicted by colored rectangles: 6xHistidine tag for purification in pale grey, thioredoxin solubilizing tag (Trx) in grey, tobacco each virus cleavage motif (ENLYFQGS) in yellow, and the linear fused proteins ASK1-SLY1 in green. **(B)** Chromatogram from gel filtration chromatography of the linear fusion of ASK1-SLY1. **(C)** SDS-PAGE corresponding to the gel filtration chromatography of linear fusion of ASK1-SLY1. A band at ~75 kDa corresponds to the endogenous E. coli HSP70 chaperone, which we frequently observe as a co-purifying contaminant under our purification conditions.

A

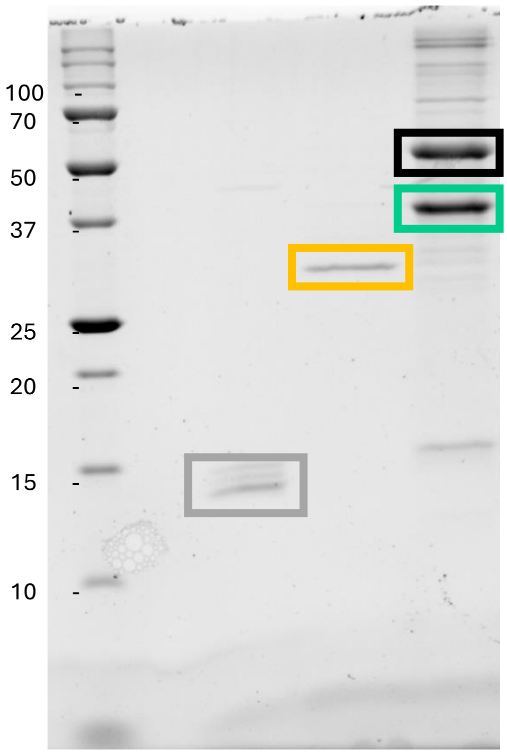

B

Custom fusion protein database search – top 3 proteins ID

N	Unused	% Coverage (95%)	Accession #	Name	Peptides (95%)
1	242.01	96.2%	6xHis-Trx-ASK1-Fusion	6xHis-Trx-ASK1-Fusion	401
2	60	76.1%	cont|000137	spt|P00760| Cationic trypsin ...	86
3	6	5.7%	cont|000135	cra|hCP1609934.2| keratin 1 ...	3

Trx-ASK1-SLY1 fused protein sequence coverage (blue)

MGSSHHHHHHSSGTSDKIIHLTDDSFDTDVLKADGAILVDFWAE
WCGPCKMIAPILDEIADEYQGKLTVAKLNIDQNPGTAPKYGIRG
IPTLLLFKNGEVAATKVGALSKGQLKEFLDANLAGTENLYFQGS
MSAKKIVLKSSDGESFEVEEAVALESQTIAHMVEDDCVDNGVP
LPNVTSKILAKVIEYCKRHVEAAASKAEAVEGAATSDDDLKAWD
ADFMKIDQATLFELILAANYLNIKNLLDLTCQTVADMIKGKTPEEI
RTTFNIKNDFTPEEEEEVRRENQWAFEGSMKRSTTDSDLAGDA
HNETNKKMKSTEEEEIGFSNLDENLVYEVLKHVDAKTLAMSSC
VSKIWHKTAQDERLWELICTRHWTNIGCGQNQLRSVVLALGG
FRRLHSLYLWPLSKPNPRARFGKDELKLTLSLLSIRYYEKMSFTK
RPLPESK

Figure S7. Mass spectroscopy analysis of the ASK1-SLY1 fusion. (A) SDS-PAGE analysis of samples obtained by gel-filtration purification of TEV-cleaved linear fusion of ASK1-SLY1. Black box: Purified linear fusion Trx-ASK1-SLY1 identified by LC-MS/MS. Green box: ASK1-SLY1 generated after TEV cleavage. Yellow box: TEV protease. Grey box: Thioredoxin solubility tag. **(B)** Non cleaved protein from black box was analyzed by LC-MS/MS confirming the amino acid sequence and identity of the protein of the linear fusion of ASK1-SLY1.

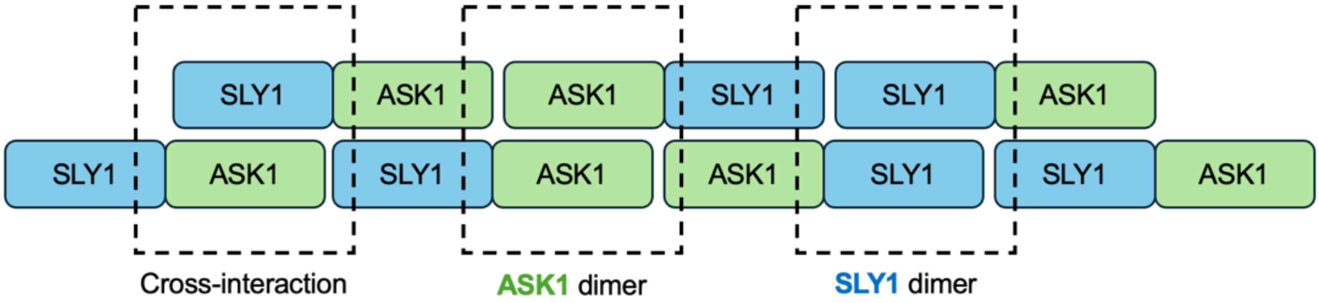

Figure S8. Schematic model of potential oligomeric superstructures formed by fused ASK1-SLY1. Three distinct interaction interfaces are depicted: (1) Cross-interaction between ASK1 and SLY1. (2) ASK1 homodimer. (3) SLY1 homodimer. Proposed oligomeric architectures are based on structural and biochemical evidence.

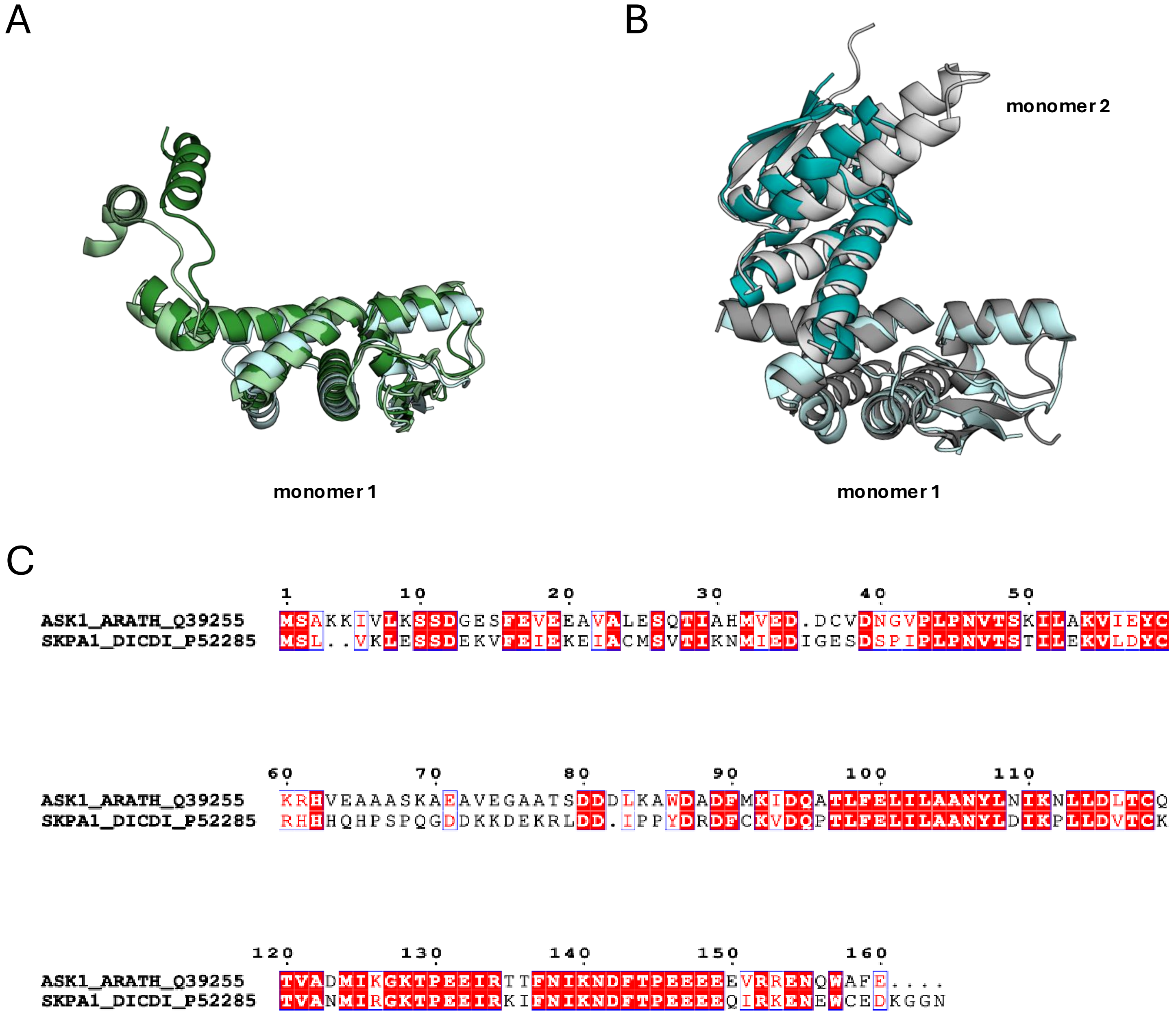

Figure S9. Alignment of DdSKPA1 structures. (A) Alignment of three-dimensional structures of monomeric SKPA1 from *Dictyostelium discoideum*, solved by NMR (light blue, PDB: 6V88), predicted by AlphaFold (forest green), and solved by cryo-EM (light green, PDB: 9O4K). **(B)** Alignment of dimeric structures: the NMR structure of DdASKPA1 (turquoise, residues 1–116) and the AlphaFold prediction of AtASK1 (gray, residues 1–127). **(C)** Sequence alignment of DdSKP1 and AtASK1.

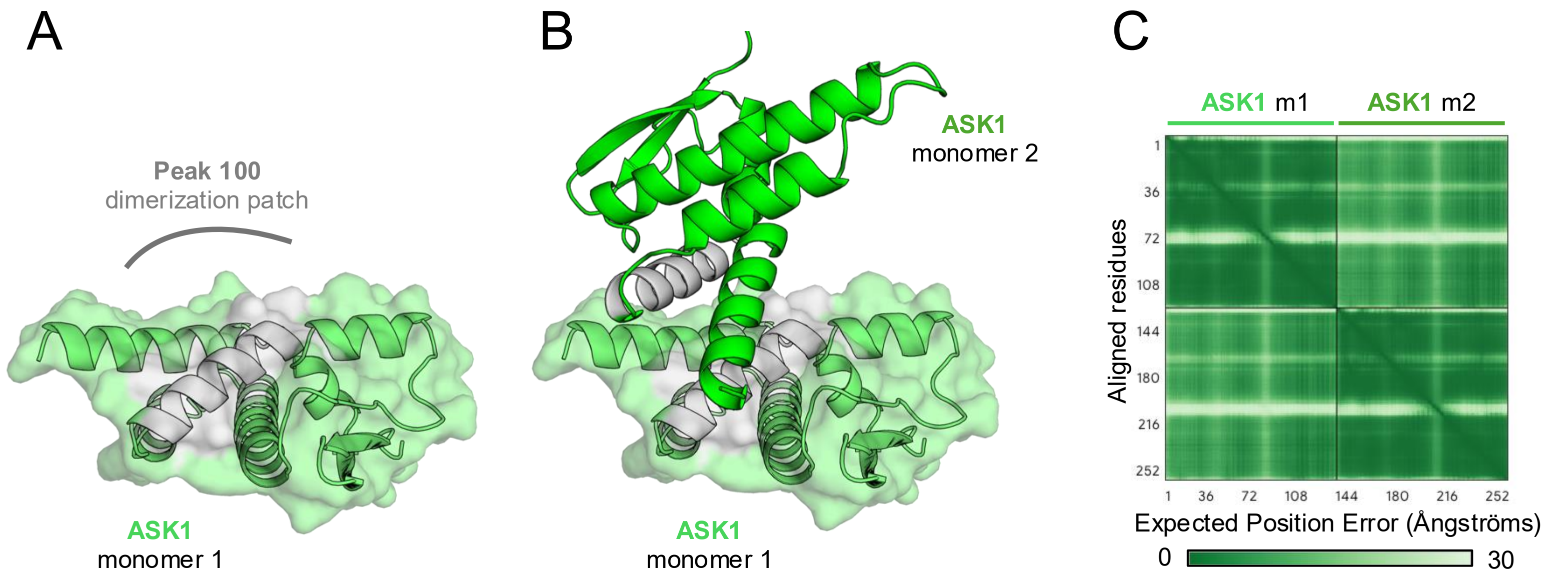

Figure S10. Predicted dimerization of AtASK1. (A) Predicted three-dimensional structure of AtASK1. The protein is shown in light green on cartoon and surface representations, and the hydrophobic patch predicted by the Kyte-Doolittle scale is colored in grey on helix α5 and labeled "dimerization patch". **(B)** Predicted three-dimensional structure of an AtASK1 dimer complex. Monomer 2 is shown in dark green, and the hydrophobic patch is highlighted in grey on the second α5 helix. **(C)** The predicted aligned error (PAE) diagram obtained with AlphaFold v3 suggests an interaction between two monomers of AtASK1.

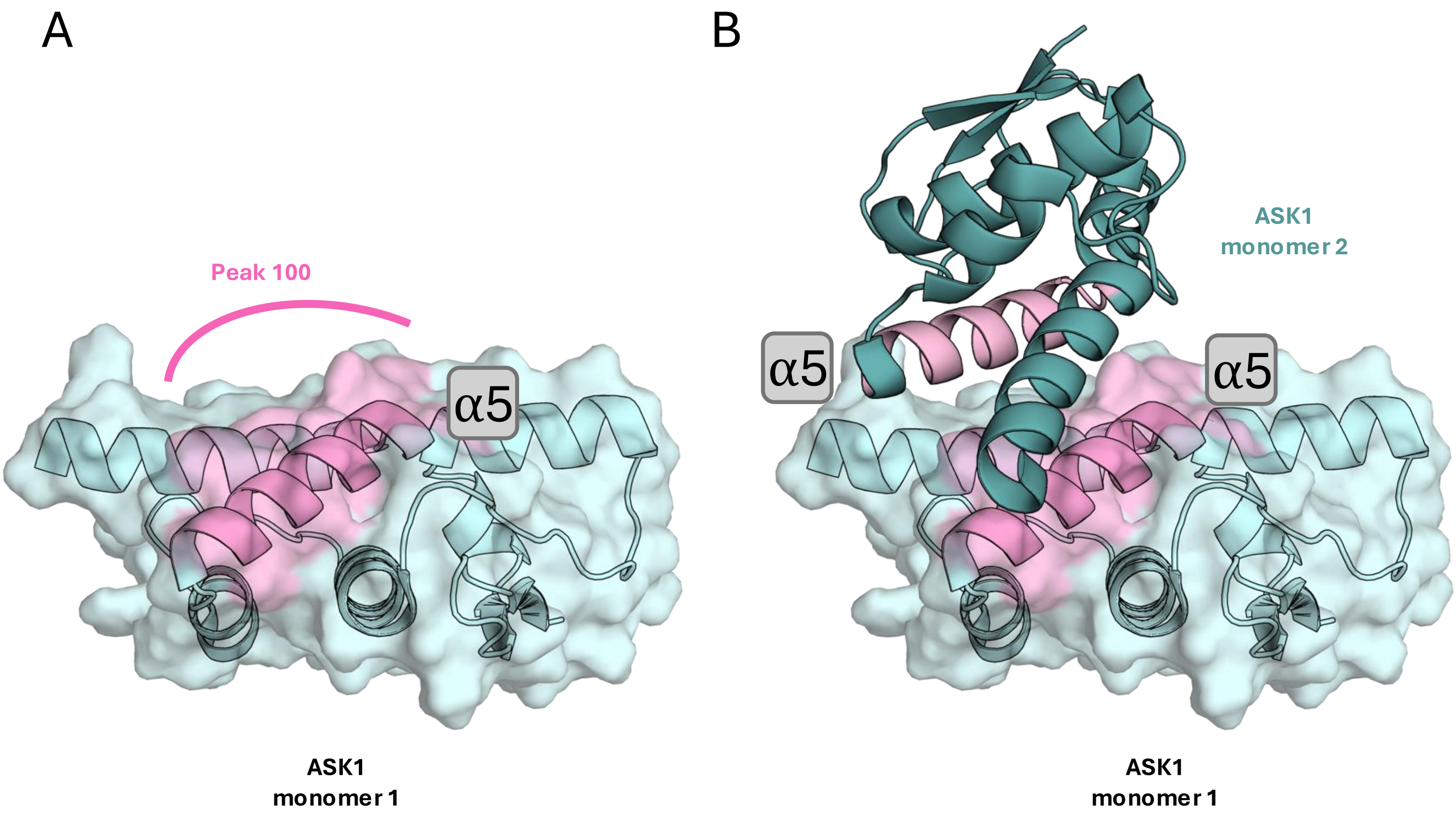

Figure S11. Dimerization of SKPA1 from *Dictyostelium discoideum*. (A) NMR structure of a monomeric SKPA1 (PDB: 6V88). The protein is shown in light green, cartoon, and surface representation, while the hydrophobic patch predicted by the Kyte-Doolittle scale is depicted in mauve on helix α5 and labeled as "dimerization patch" corresponding to the putative dimerization surface. **(B)** NMR structure of the DdSKPA1 dimer. The monomer is shown in teal, and the hydrophobic patch is highlighted in mauve on the second α5 helix.

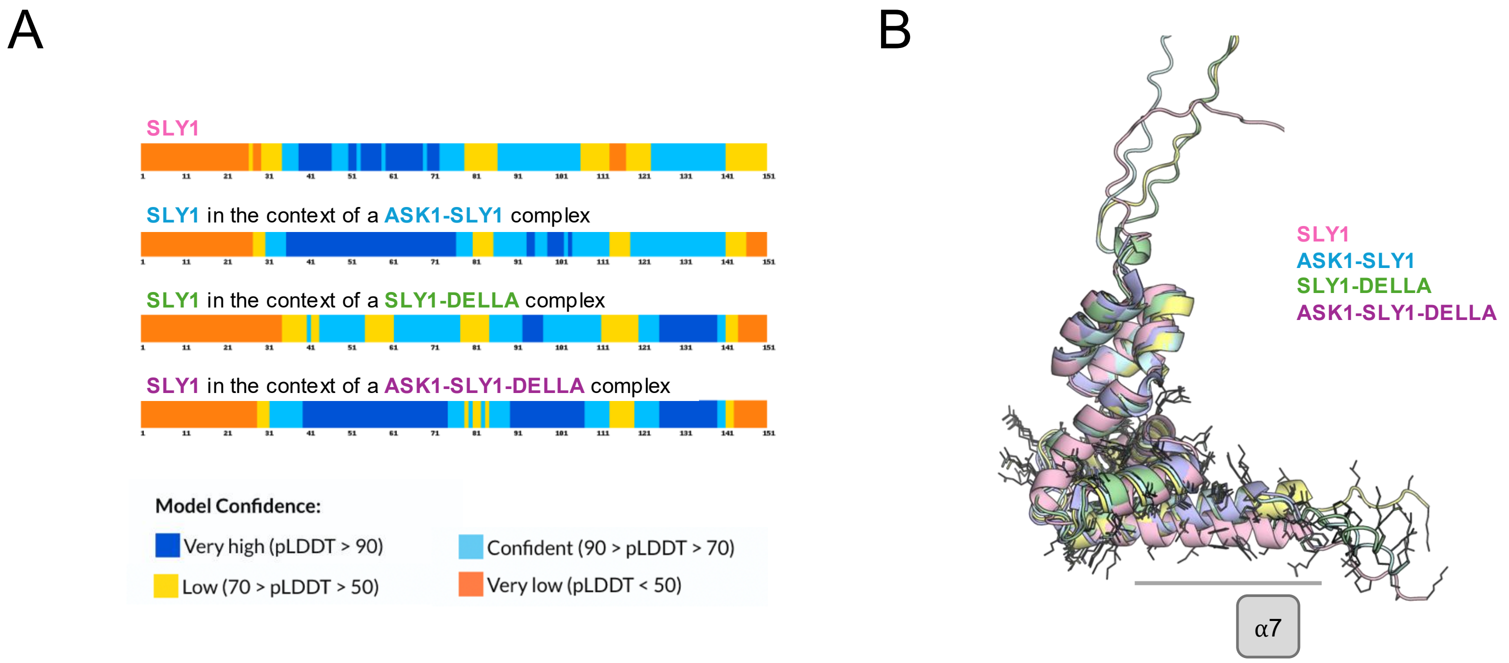

Figure S12. Structural consequence of SLY1 binding to ASK1 or DELLA. (A) pLDDT diagrams for SLY1 modeled alone or in complex with ASK1 or DELLA. SLY1 proteins are represented by rectangles, colored according to their per-residue pLDDT values. **(B)** Structural alignment of AlphaFold-predicted structures of SLY1 in apo or complexed forms along with the cryo-EM structure depicted in yellow (PDB: 9O4K).

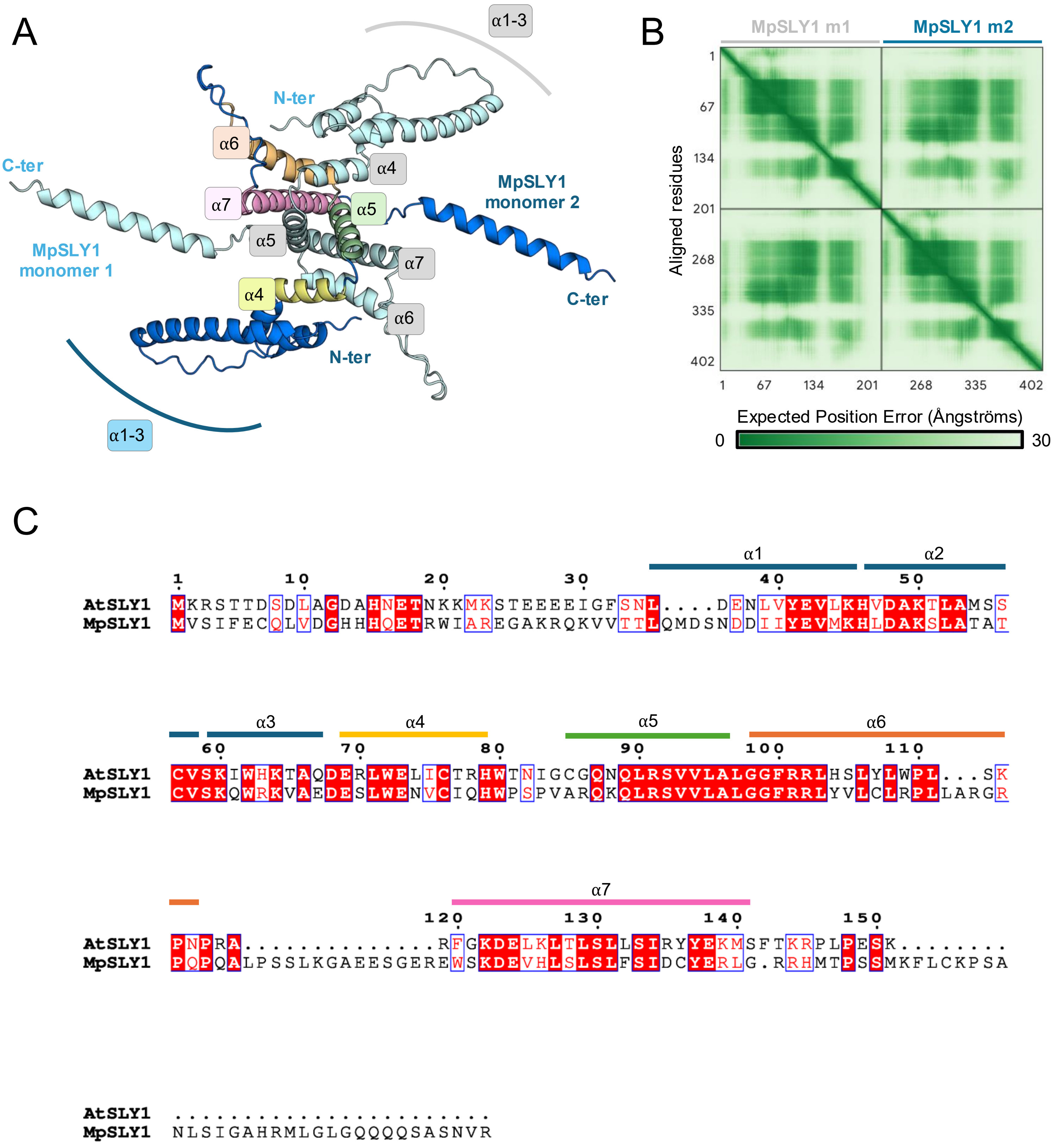

Figure S13. Predicted dimeric structures of MpSLY1. (A) Structure of a MpSLY1 dimer predicted with AlphaFold v3. **(B)** Predicted aligned error (PAE) diagram for the MpSLY1 dimeric structure shown in **(A).** The PAE diagram suggests an interaction between two monomers of AtASLY1. **(C)** Alignment of SLY1 from *Arabidopsis thaliana* and *Marchantia polymorpha*. The position of the α-helices of AtSLY1 are indicated on top of the alignment.

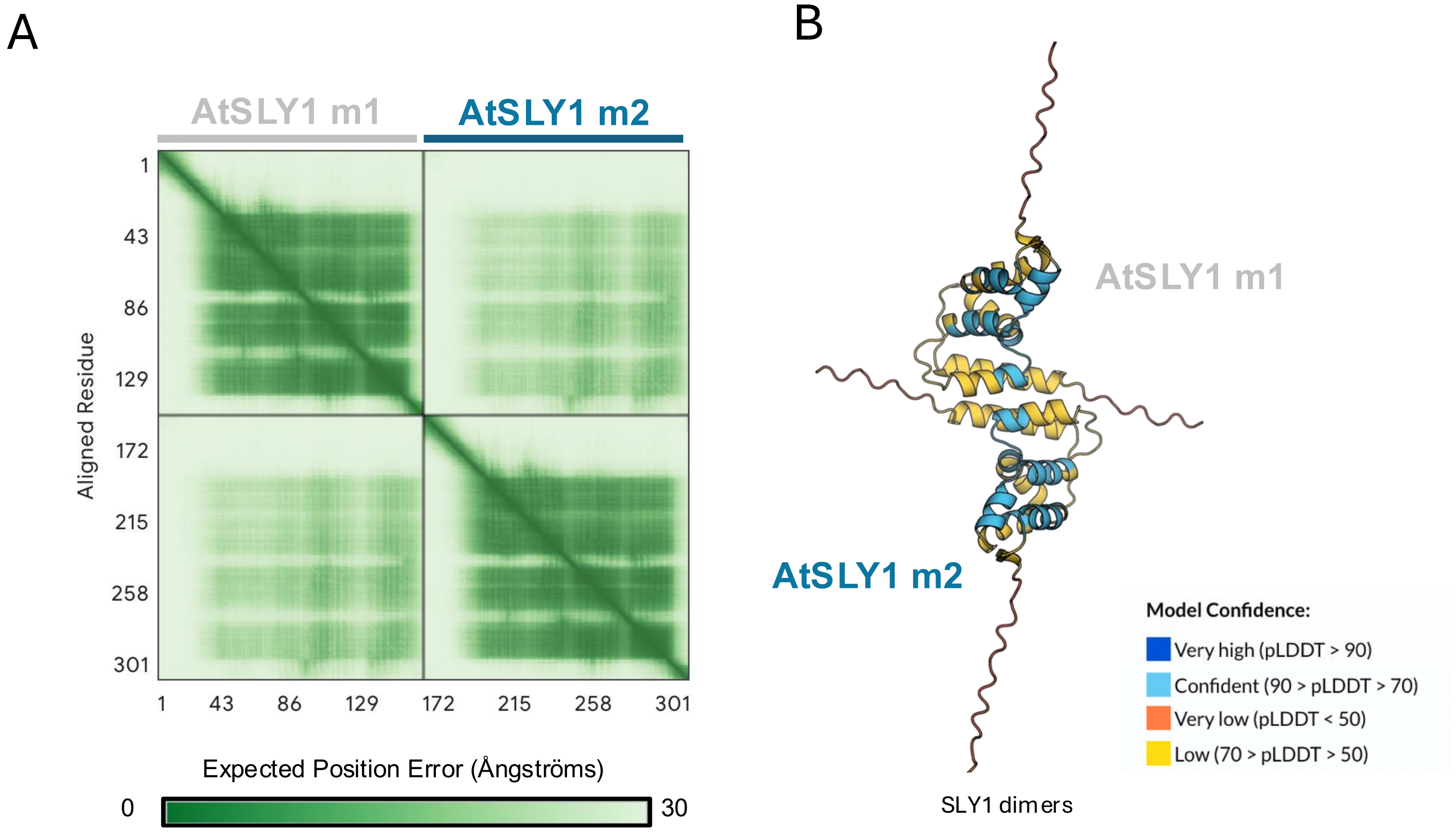

Figure S14. AlphaFold-predicted structures of AtSLY1 dimers. (A) Predicted aligned error plot (PAE) obtained by AlphaFold v3 suggests **(B)** an interaction between 2 monomers of AtASLY1.

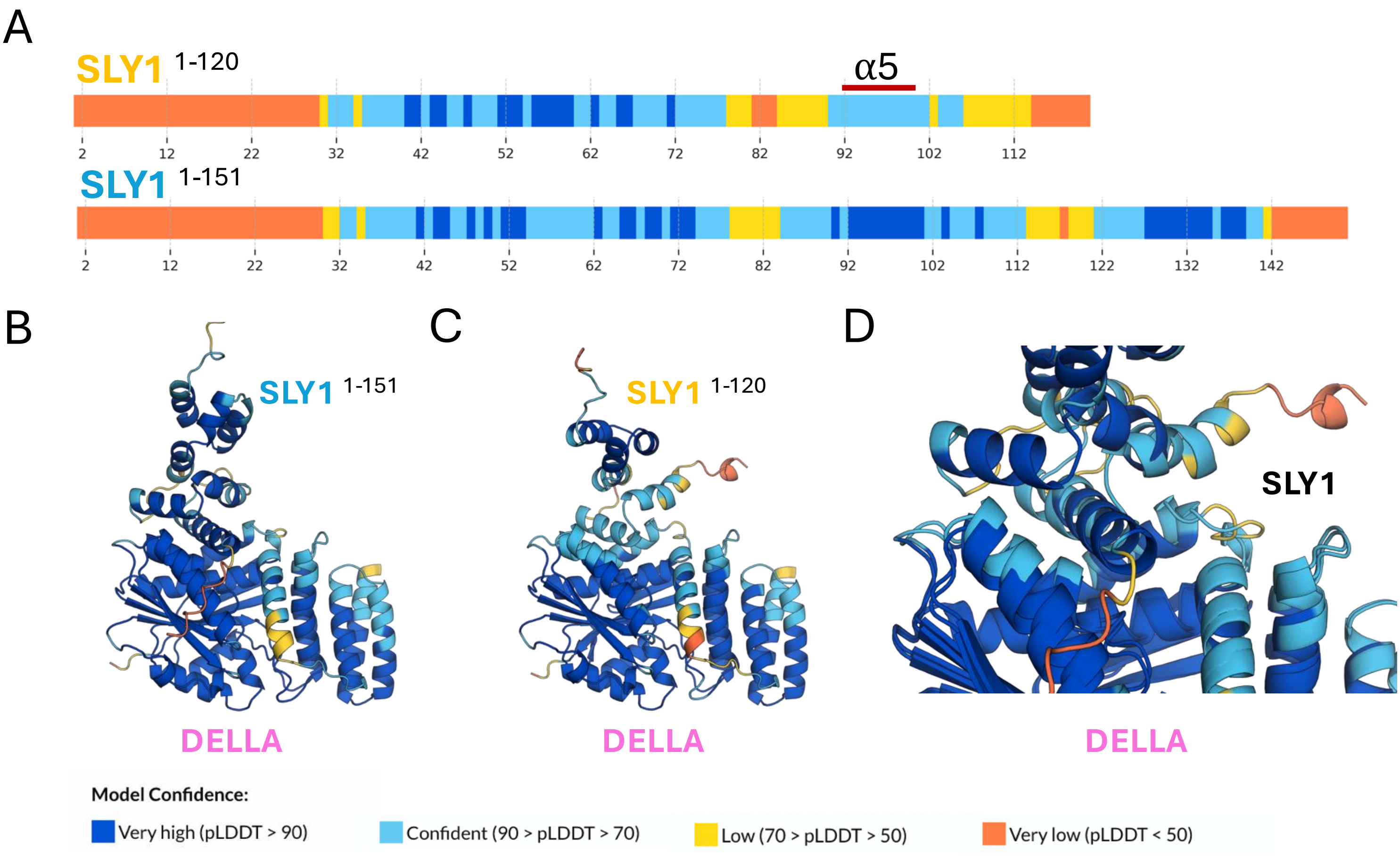

Figure S15. Structural comparison of SLY1$^{1\text{-}151}$-RGL1^{WT} and SLY1$^{1\text{-}120}$-RGL1^{WT} complexes generated by AlphaFold. **(A)** AtSLY1$^{1\text{-}151}$ and SLY1$^{1\text{-}120}$ are represented by rectangles, colored according to their per-residue pLDDT values. **(B)** AtSLY1$^{1\text{-}151}$ / AtRGL1^{WT}. **(C)** AtSLY1$^{1\text{-}120}$ / RGL1^{WT}. AlphaFold predicted interaction structure between SLY1, and DELLA are colored according to their per-residue pLDDT values. **(D)** Close up view on the alignment of SLY1-$^{1\text{-}151}$ and SLY1 $^{1\text{-}120}$ interacted with DELLA.
